# The Antioxidant *Carrichtera annua* DC. Ethanolic Extract Counteracts Cisplatin Triggered Hepatic and Renal Toxicities

**DOI:** 10.3390/antiox10060825

**Published:** 2021-05-21

**Authors:** Enas E. Eltamany, Sameh S. Elhady, Mohamed S. Nafie, Haidy A. Ahmed, Dina M. Abo-Elmatty, Safwat A. Ahmed, Jihan M. Badr, Asmaa R. Abdel-Hamed

**Affiliations:** 1 Department of Pharmacognosy, Faculty of Pharmacy, Suez Canal University, Ismailia 41522, Egypt; enastamany@gmail.com (E.E.E.); haidyabdelkader@gmail.com (H.A.A.); safwat_ahmed@pharm.suez.edu.eg (S.A.A.); 2 Department of Natural Products, Faculty of Pharmacy, King Abdulaziz University, Jeddah 21589, Saudi Arabia; ssahmed@kau.edu.sa; 3 Department of Chemistry, Faculty of Science, Suez Canal University, Ismailia 41522, Egypt; mohamed_nafie@science.suez.edu.eg; 4 Ismailia Health Affairs Directorate, Ismailia 41525, Egypt; 5 Department of Biochemistry, Faculty of Pharmacy, Suez Canal University, Ismailia 41522, Egypt; dinawahadan@yahoo.com (D.M.A.-E.); asmaa.ramdan@pharm.suez.edu.eg (A.R.A.-H.)

**Keywords:** *Carrichtera annua*, cisplatin, hepatotoxicity, nephrotoxicity, phenolic acids, flavonoids, molecular docking

## Abstract

Cisplatin is a powerful anti-neoplastic drug that displays multi-organ toxicity, especially to the liver and kidneys. Consumption of phytomedicines is a promising strategy to overcome the side effects of chemotherapy. *Carrichtera annua* extract proved to possess potent antioxidant activity. Its protective potential against cisplatin-induced hepato–nephrotoxicity was scrutinized. Moreover, a phytochemical study was conducted on *C. annua* ethyl acetate fraction which led to the isolation of five known phenolic compounds. Structure determination was achieved utilizing ^1^H- and ^13^C-NMR spectral analyses. The isolated phytochemicals were *trans*-ferulic acid (**1**), kaempferol (**2**), *p*-coumaric acid (**3**), luteolin (**4**) and quercetin (**5**). Regarding our biological study, *C. annua* has improved liver and kidney deteriorated functions caused by cisplatin administration and attenuated the histopathological injury in their tissues. Serum levels of ALT, AST, blood urea nitrogen and creatinine were significantly decreased. *C. annua* has modulated the oxidative stress mediated by cisplatin as it lowered MDA levels while enhanced reduced-GSH concentrations. More importantly, the plant has alleviated cisplatin triggered inflammation, apoptosis via reduction of INFγ, IL-1β and caspase-3 production. Moreover, mitochondrial injury has been ameliorated as remarkable increase of mtDNA was noted. Furthermore, the MTT assay proved the combination of cisplatin—*C. annua* extract led to growth inhibition of MCF-7 cells in a notable additive way. Additionally, we have investigated the binding affinity of *C. annua* constituents with caspase-3 and IFN-γ proteins using molecular simulation. All the isolated compounds exhibited good binding affinities toward the target proteins where quercetin possessed the most auspicious caspase-3 and IFN-γ inhibition activities. Our results put forward that *C. annua* is a promising candidate to counteract chemotherapy side effects and the observed activity could be attributed to the synergism between its phytochemicals.

## 1. Introduction

Cisplatin (cis-diaminedichloroplatinum (II)) is an efficient antineoplastic drug that is extensively utilized in treatment of several malignancies, including tumors of the lungs, bladder, stomach, ovaries, cervix, testes and many other organs [1]. The anticancer effect of cisplatin is achieved by mitosis blockage and induction of apoptosis and necrosis of malignant cells [2]. However, its lack of selectivity represents a crucial problem as it interacts in the same way with rapidly dividing normal healthy cells, resulting in severe often life-threatening side effects.

Cisplatin sparks reactive oxygen species (ROS) accumulation and the depletion of antioxidant defenses in the tissues generating an oxidative stress state. ROS trigger protein denaturation, peroxidation of membrane lipids and DNA damage inflicting injury and necrosis of cells [3]. These are the key factors for initiating cisplatin life-threatening adverse effects including neurotoxicity [4], nephrotoxicity [5,6,7,8,9,10] and hepatotoxicity [8,9,10,11,12].

Cisplatin inflicts hepatotoxicity via stimulating apoptosis, disturbance of Ca^2+^ homeostasis and mitochondrial injury, in addition to overexpression of pro-inflammatory genes such as inducible nitric oxide synthase and COX-2, which have a key role in the mechanism mediating hepatotoxicity induced by cisplatin [13].

Regarding the nephrotoxicity unleashed by cisplatin, several mechanisms have been suggested, including oxidative stress, inflammation, mitochondrial dysfunction, DNA adducts and direct cytotoxicity to the renal tubules’ epithelial cells which will lead to acute kidney injury (AKI) [14,15]. About 20–30% of patients administered cisplatin-based treatment regimens end up suffering from acute kidney injury (AKI) [15,16]. Chronic or end-stage renal disease are fatal complications of AKI with socioeconomic impact. Dialysis and kidney replacement are the only treatments of end-stage renal disease [15]. Until now, there is no clinically curative or preventive drug for cisplatin-caused nephrotoxicity [16]. The symptomatic therapies of AKI embracing saline hydration, mannitol-based osmotic diuresis, and drug discontinuation [15,17]. However, forced diuresis by mannitol inflicts over-diuresis leading to dehydration in patients undergoing cisplatin chemotherapy [17]. Moreover, in some trials, mannitol administration has been associated with increased rates of renotoxicity. Besides, the use of forced diuresis for the prevention of cisplatin-induced nephrotoxicity is not recommended by the European Society of Clinical Pharmacy Special Interest Group on Cancer Care [18]. Hence, the discovery of safe and potential adjuvant therapy is need urgently for high dose cisplatin-receiving patients.

Herbal medicine and plant derived antioxidants are recognized as a successful strategy for ameliorating cisplatin-induced toxicities [6,19]. Numerous traditionally used herbs were reported to ameliorate cisplatin’s adverse effects, especially hepato- and reno-toxicities, such as *Dendropanax morbiferatea* [19], ginger [20], marjoram [6], grape seed [21] and pomegranate rind [22]. Volatile oils of nigella [23], fennel [24] and *Pituranthos chloranthus* [25] have been proven to counteract cisplatin-induced renal and hepatic toxicities. Moreover, various phytochemicals demonstrated reno-and hepato-protective effects such as linalool [7], thymoquinone [26], cinnamic acid and its derivatives [10,27,28,29] as well as some flavonoids [30,31,32]. The protective effect of phenolic acids and flavonoids against cisplatin toxicity could be explained on the basis of their distinctive antioxidant properties. These unique compounds have various ROS-scavenging modes as well as the ability to act as electron or hydrogen donors, reducing and metal chelating agents. Such properties are ascribed to the varying numbers of hydroxyl groups in their structures and different conjugation patterns [33].

*Carrichtera annua* DC. (Cruciferae) is an edible plant [34]. It grows in various regions in Egypt; for instance, the Sinai Peninsula, the Nile Delta and the North Coast [35]. In our previous study, *C. annua* was proved to possess auspicious antioxidant and anticancer activities [36] which are attributed to its unique plethora of phytoconstituents, particularly phenolics and flavonoids [35,36,37,38,39,40].

Based on the abovementioned considerations and encouraged by the reported antioxidant potential of *C. annua* crude extract which is correlated to its phenolics and flavonoids content [36], the plant was chosen for our present investigation. Since there is hardly any data in the literature on the effect of *C. annua* against cisplatin-induced hepato- and reno-toxicities therefore, our study was directed towards the assessment of the hepato- and reno-protective potentials of *C. annua* crude extract using an in vivo model. Moreover, a phytochemical investigation of *C. annua* ethyl acetate fraction was conducted to isolate the compounds that may contribute to its protective effect. Their interactions with caspase-3 and interferon gamma were scrutinized by molecular docking studies.

## 2. Materials and Methods

### 2.1. Chemicals and Instruments

The ^1^H (400 MHz) and ^13^C (100 MHz)-NMR spectra were recorded by on a JEOL (Freising, Germany) spectrometer using CD_3_OD and DMSO-d_6_ as solvents and tetramethylsilane (TMS) as an internal standard. For column chromatography: normal-phase silica gel 60 (230–400 mesh, Merck KGaA, Darmstadt, Germany) and Sephadex LH-20 (Sigma Aldrich^®^ a subsidiary of Merck KGaA, Darmstadt, Germany) were used. Merck precoated silica gel F254 plates were used for analytical TLC purpose. SIL G-25 Unmodified Standard Silica Layer Glass Plates with thickness of 2 mm (Macherey-Nagel^®^, Düren, Germany) were used for preparative TLC. Visualization of TLC was carried out using UV light and anisaldehyde—sulphuric acid spray reagent. Cisplatin (Cisplatine^®^) was obtained from Mylan Company (Saint Priest, France). Phosphate buffered saline solution (PBS) was purchased from Lonza Bio-products (Verviers, Belgium).

### 2.2. Plant Material Collection and Extraction

*Carrichtera annua* DC. (Cruciferae) aerial parts were collected from the south of the Sinai Peninsula (Egypt) in June 2014. Authentication of the plant was performed by Prof. Dr. Elsayeda M. Gamal El-Din (Department of Botany, Faculty of Science, Suez Canal University, Ismailia, Egypt). A voucher specimen of *C. annua* (Registration number SAA-159) was placed in the Herbarium of Pharmacognosy Department, Faculty of Pharmacy, Suez Canal University. The collected plant was dried at room temperature and then pulverized. Then, two kilograms of powdered aerial part of *C. annua* were extracted with ethanol till exhaustion. The extracts were combined, dried under vacuum at 40 °C to give 72.9 g of brownish-green residue.

### 2.3. Animals

Thirty adult male Wister rats weighing 180–200 g were obtained from National Research Institute (Cairo, Egypt). Animals were accommodated in an animal room under controlled temperature (22–24 °C) and maintained at 12 h light–dark cycles. Rats were provided with standard rat chow and tap water ad libitum. The experiment has begun after an adaptation period of one week. In accordance with the Guide for the Care and Use of Laboratory Animals (National Research Council, 2011, the experimental animals were kept, handled and used. All experimental protocols received the approval of the Ethics Committee at the Faculty of Pharmacy, Suez Canal University (code # 202001R2).

### 2.4. Experimental Design

Rats were distributed equally into three groups (10 rats per group) and the experiment was continued for 2 weeks. Group 1 was the normal control group; rats received appropriate volumes of normal saline. Group 2 was the cisplatin group; rats were injected intraperitoneally with cisplatin (8 mg/kg) once at 12th day of the experiment. Group 3 received a daily dose of *C. annua* extract (250 mg/kg, orally) for 14 days and a single dose of cisplatin (8 mg/kg, i.p.) at 12th day of the study according to [7].

### 2.5. Blood and Tissue Sampling and Biochemical Analysis

After the experiments were finished, rats were injected with urethane (1.2 g/kg; i.p.) for anesthesia. Blood samples were withdrawn from retro-orbital plexus, serum and plasma were separated and kept at −80 °C. The animals were euthanized then kidney and liver were excised washed with ice phosphate buffer saline and dried using filter paper. The liver and kidney of each rat were divided in two parts: a part was stored at −80 °C until performing the biochemical measurements and the other was fixed in 10% neutral buffered formalin for immunohistochemical and histopathological investigations.

#### 2.5.1. Histopathological Examination

After fixation in 10% neutral buffered formalin, liver and kidney tissues were dehydrated with ethanol then embedded into paraffin wax blocks. Sections of approximately 5 µm were prepared then stained with hematoxylin/eosin (H&E) according to the method mentioned [41] then blindly examined for the extent of liver and kidney damage. The obtained sections were photographed by means of standard digital microscope camera (CX 41 binocular microscope, Olympus Deutschland, Hamburg, Germany).

#### 2.5.2. Liver Function Tests

Serum levels of aspartate aminotransferase (AST) and alanine aminotransferase (ALT) were estimated colourimetrically using a Biodiagnostic kit (Giza, Egypt) according to [42] and manufacturer’s instructions. In brief, 0.5 mL of ALT or AST buffer substrate was incubated for 5 min at 37 °C and mixed with 0.1 mL of serum and the mixture was incubated again at 37 °C for 60 min in case of AST and 30 min in case of ALT. Then, 0.5 mL of color reagent (2,4 dinitrophenylhydrazine) was added and the mixture was left for 20 min. at room temperature then mixed with 5 mL of NaOH (0.4 N). After 5 min, the absorbance was measured at λ 505 nm.

#### 2.5.3. Kidney Function Tests

To evaluate the kidney functions, serum creatinine level was measured according to the method adapted by Murray [43]. The method is based on the formation of a colored complex between creatinine and picric acid in alkaline medium. Briefly, equal volumes of picric acid (20 mmol/L) and sodium hydroxide (1.2 mmol/L) were mixed immediately before the assay to obtain the working reagent. Then, 0.5 mL of serum were mixed with 0.5 mL of the working solution and incubated for 5 min. at 37 °C. The absorbance was recorded at λ 520 nm. 

Besides, blood urea nitrogen (BUN) was estimated using the method mentioned in [44] by enzymatic colorimetric methods using a Biodiagnostic kit following the procedure provided by the manufacturer. The method depends on the production of ammonia from urea by the action of urease enzyme. The ammonium ions produced are estimated by the Berthelot reaction to obtain blue dye indophenol derivative. The intensity of the produced color is proportional to initial urea concentration. In brief, about 10 µL of serum was mixed with 200 µL of buffer—enzyme solution which consisted of phosphate buffer (50 mmol/L) and urease enzyme (>10,000 µ/L). The mixture was incubated 5 min. at 37 °C and 1 mL of color reagent (phenol (100 mmol/L) and sodium nitroprusside (0.2 mmol/L)) was added followed by the addition of 1 mL of alkaline reagent (composed of 150 mmol/L of sodium hydroxide and 15 mmol/L sodium hypochlorite). After incubation for 10 min. at 37 °C, the absorbance was estimated at λ 550 nm.

#### 2.5.4. Estimation of Malondialdehyde (MDA) and Reduced Glutathione (GSH)

Tissue malondialdehyde (MDA) level was estimated spectrophotometrically according to method described by Ohkawa and coworkers [45]. The method depends on the reaction of MDA with thiobarbituric acid and 1,1,3,3-tetramethoxypropane as was employed as a standard. In brief, about 0.1 g of tissue was homogenized at 4 °C in 1 mL of sodium phosphate buffer (0.1 M, pH 7.4) containing EDTA (0.1 mM). Then, 1ml of the homogenate was added to 2ml of thiobarbituric acid (TBA) reagent (composed of 15% trichloroacetic acid (TCA), 0.375% TBA, and 0.25 N HCl). The mixture was boiled for 15 min then ice-cooled, and centrifuged for 10min. at 3500 rpm. Using a UV-visible spectrophotometer (UV-1601PC, Shimadzu, Tokyo, Japan) the color intensity of the supernatant was recorded.

Tissue reduced glutathione (GSH) contents were estimated according to the method adapted by Ellman [46]. Briefly, about 0.5 mL of tissue homogenate in phosphate buffer was added to an equal volume of 10% trichloroacetic acid. 6 mM EDTA and the mixture was shaken gently for 10–15 min then centrifuged at 2000 rpm for 5 min. Then 0.2 mL of the supernatant was added to 1.7 mL of phosphate buffer (0.1 M, pH 8) followed by the addition of 0.1 mL of Ellman’s reagent (0.039 g of 5,5′-dithio-bis-(2nitrobenzoic acid) dissolved in 10 mL Phosphate buffer) The absorbance of the reaction product was recorded after 5 min. at λ 412 nm using a Shimadzu spectrophotometer.

#### 2.5.5. Determination of Interferon Gamma, Interleukin-1β and Caspase 3

Serum levels of the inflammatory marker interferon gamma was determined using Rat Interferon Gamma (IFN-γ) ELISA Kit (Cat No. MBS267008) (Biosource International, Foster city, CA, USA) according to manufacturer’s instructions. In brief, this assay applied double-sandwich elisa method and the provided ELISA Kit is typical. The pre-coated antibody used is Rat IFN-γ monoclonal antibody while the polyclonal antibody with biotin labeled served as the detecting antibody. Both of biotin labeling antibody and samples were added into ELISA plate wells then rinsed out PBS or TBS. After that, Avidin-peroxidase conjugates were placed in ELISA wells in order; TMB substrate was added for coloring after reactant was rinsed by PBS or TBS. TMB acquired a blue color in peroxidase catalytic and finally became yellow by the action of acid. The testing factors in samples and the color intensity are directly correlated.

Serum levels of the apoptotic marker caspase 3 was determined using Rat Caspase-3 (Caspase-3) ELISA Kit (Cat No. MBS261814) (Biosource International) according to the manufacturer’s instructions. This experiment, a double-sandwich elisa procedure was applied and the provided ELISA Kit is typical. Rat Caspase-3 monoclonal antibody was served as the pre-coated antibody while the polyclonal antibody with biotin labeled was the detecting antibody. Samples as well as biotin labeling antibody were planed into ELISA plate wells then rinsed with PBS or TBS. After that, Avidin-peroxidase conjugates were put onto ELISA wells in order; TMB substrate was applied as coloring reagent after the reactant was washed out by PBS or TBS. TMB acquired a blue color in peroxidase catalytic which finally became yellow by the addition of an acid. The testing factors in samples and the color depth were directly proportional.

Furthermore, immunohistochemical investigation was performed to evaluate the activity of the inflammatory and apoptosis biomarkers interleukin-1β (IL-1β) and caspase-3 respectively. Sections from liver and kidney tissues were stained with anti-IL-1β (Catalog number bs-0812R, Bioss Inc., Woburn, MA, USA) and anti-caspase 3 (Catalog number GTX30246, GeneTex, Irvine, CA, USA) after preparation according to the method described in [41,47]. 3,3-diaminobenzidine (Power-Stain™ 1.0 Poly HRP DAB Kit for Mouse + Rabbit, GENEMED, South San Francisco, CA, USA) was applied as a chromogen for immunoreaction visualization, as well as Mayer’s haematoxylin as a counterstaining. Selected parts of liver and kidneys were photographed by a digital camera (Olympus Dp25). Then, the obtained images were inspected utilizing image analysis software (ImageJ version 1.53 h) according to the method described by Elgawish and coworkers [48] where the IHC stained area percentages were determined using the following equation:

%IHC stained area = IHC stained area/total area × 100(1)

#### 2.5.6. Isolation of Mitochondria and Mitochondrial DNA

By differential centrifugation, mitochondria were extracted according to the procedure reported by Chappel and Hansford [49]. In brief, the tissue sample was homogenized in Tris–sucrose (composed of a solution of 0.25 M sucrose in 0.7 M Tris–HCl Buffer (pH 7.4). To assist the disruption of cells, EDTA was added. Then, the produced homogenate was centrifugated at 2500× *g* for 10 min for nuclei and unbroken cells removal. To form primary mitochondrial pellet, the supernatant fluid obtained from the previous step was decanted then spinned at 10,000× *g* for 10 min. After supernatant decantation, the produced pellet was washed by suspension in Tris–sucrose then centrifugation. The previous step was repeated to ensure the mitochondrial purity. At last, the mitochondrial pellet is re suspended (1 mL Tris–sucrose/1 g of original sample). Isolation of mitochondrial DNA (mtDNA) was carried out using the alkaline denaturation method performed as outlined in [50]. Quantification of DNA and determination of its purity were achieved by means of a NanoDrop™ 1000 Spectrophotometer V.3.7 (Thermo Fisher Scientific Inc., Wilmington, DE, USA).

### 2.6. In Vitro Anticancer Assay of C. annua—Cisplatin Combination

The anticancer potential of *C. annua* extract combination with cisplatin was evaluated on MCF-7 breast cancer cell line using MTT assay according to the method described in [8,36]. In brief, MCF-7cells were kept in RPMI-1640/DMEM (Sigma-Aldrich, St. Louis, MO, USA) supplemented with 10% FBS (Sigma, St. Louis, MO, USA), 2 mM L-glutamine (Lonza, Bornem, Belgium) and 1% penicillin/streptomycin (Lonza). Cells were incubated in 5% CO_2_ atmosphere (NuAire) at 37 °C according to the routine procedure [51]. MCF-7 Cells were plated in triplicates in a 96-well plate at a density of 5000 cell and incubated for 24 h. Stock solutions (1 mg/mL) were prepared in deionized water of cisplatin and *C. annua*—cisplatin combination both at the same concentrations. Then, the MCF-7 cells were treated with test samples at the specified concentrations in 100 μL media as a final volume. After 72 h, cell viability was evaluated by adding 20 μL of MTT solution (Promega, Madison, WI, USA) [52] to each well. In this test, tetrazolium rings of the MTT dye (yellow water-soluble substrate) were cleaved into dark blue formazan crystal, which were solubilized in DMSO. The plate was incubated for 3 h. Using a plate reader, the fluorescence was subsequently estimated at λ 570 nm and IC_50_ values were calculated applying the GraphPad Prism 7 (GraphPad Software, San Diego, CA, USA).

### 2.7. Statistical Analysis

Data were managed using the Statistical Package for Social Sciences version 17.0 software (SPSS Inc., Chicago, IL, USA). The results were expressed as mean ± SE. One-way analysis of variance, ANOVA, followed by Bonferroni’s multiple comparisons test were employed for statistical analysis. A value of *p* < 0.05 was considered to be statistically significant.

### 2.8. Fractionation of C. annua Extract and Isolation of Its Phytochemicals

A phytochemical study on *C. annua* was conducted in order to isolate its major phytoconstituents which may contribute to its chemoprotective effect. A sixty grams of the crude alcoholic extract were suspended in distilled water then fractionated by partitioning between *n*-hexane, chloroform, ethyl acetate, and *n*-butanol. The obtained fractions were dried under reduced pressure. The ethyl acetate fraction exhibited seasonable yield and promising TLC pattern therefore, it was selected for further fractionation and purification to identify its main chemical constituents. About 11.6 g of *C. annua* ethyl acetate fraction was chromatographed on a normal phase silica gel column (30 × 4.5) eluted initially with 100% CHCl_3_, followed by gradients of CHCl_3_: MeOH and culminating with CHCl_3_: MeOH (1:1) to afford eight subfractions (E1–E8). Subfraction (E2) (1.9 g) eluted with CHCl_3_: MeOH (95:5) was further chromatographed on a silica gel column using CHCl_3_ and MeOH gradients (from 100% CHCl_3_ to CHCl_3_: MeOH (85:15) to yield five subfractions (E2-S1 to E2-S5). Subfraction E2-S2 was subjected to gel filtration on Sephadex LH-20 eluted with CHCl_3_: MeOH (1:1) to afford compound **1** (12.4 mg, white amorphous powder). Subfraction E2-S3 was purified using Sephadex LH-20 column using CHCl_3_: MeOH (1:1) as an eluent to afford compound **2** (30.8 mg, yellow powder) and compound **3** (10.5 mg, white amorphous powder). Subfraction E2-S4 was subjected to PTLC using CHCl_3_: MeOH: HCOOH (85:14:1) as eluent to isolate two semi-pure substances A and B which were finally purified by chromatography on a Sephadex LH-20 column eluted with 100% MeOH to afford compound **4** (yellow powder, 5.5 mg) and compound **5** (yellow powder, 22 mg) respectively.

### 2.9. Spectrometric Data of the Isolated Compounds ***1**–**5***

The ^1^H and ^13^C-NMR spectral data as well as the melting point of the isolated compounds were provided in the supplementary material (Tables S1–S5 and Figures S1–S18).

### 2.10. Virtual Screening and Molecular Docking

For elucidation, the virtual mechanism of binding of all identified compounds, the molecular docking study towards the caspase-3 and IFN-γ inhibition active sites was carried out. Proteins; caspase-3 (PDB = 6CKZ) and IFN-γ (PDB: 2R3Z) were freely accessible from the PDB, their structures were optimized by adjusting the amino acids with missing atoms or alternative positions, and ligand structures were built, optimized, and energetically favored using Maestro. The molecular docking study was carried following routine work of preparation the appropriate formats of receptor and ligands, determination of grid box dimensions box of 10 Å in the x, y and z directions centered on the ligand, and finally docking with binding activities in terms of binding energies and ligand-receptor interactions, and optimized following the routine work as discussed by Nafie et al. [53] and Aly et al. [54]. MOE 2019 was employed as the validated molecular docking calculation. At last, Chimera software was utilized as the visualized software for drug target interactions assessment.

## 3. Results and Discussion

### 3.1. C. annua Protective Effects against Cisplatin Induced Hepato- and Nephro Toxicities

#### 3.1.1. Histopathological Examination

**Figure 1:**
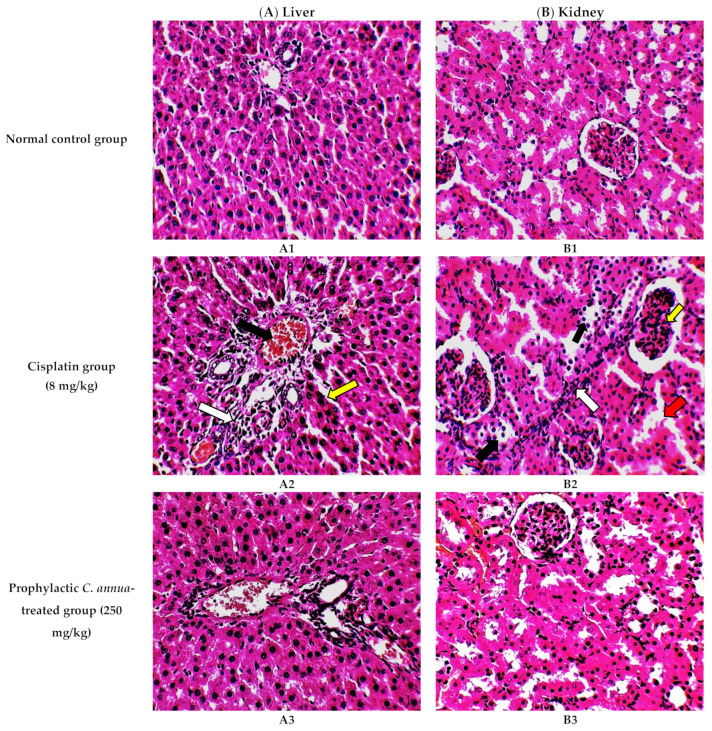
Photomicrographs of H&E- stained sections (×400) of liver (**A**) and kidney (**B**) tissues from the experimental groups indicating the protective effects of *C. annua* pretreatment on cisplatin-induced hepatic & renal toxicities. (**A1**) liver tissues of normal control group, (**A2**) liver tissues of Cisplatin group, (**A3**) liver tissues of Prophylactic *C. annua*- treated group, (**B1**) kidney tissues of normal control group, (**B2**) kidney tissues of Cisplatin group, (**B3**) kidney tissues of Prophylactic *C. annua*- treated group. In Figure 1A2 the black arrow indicates portal area congestion, the white arrow represents infiltration of inflammatory cells accompanied by tissue necrosis and the yellow arrow shows the dilatated sinusoid. In Figure 1B2, the black arrow indicates renal tubules hydropic degeneration and dilatation, the red arrow shows dilatated necrotic tubules, the white arrow represents infiltration of inflammatory cells and the yellow arrow shows glomerular atrophy.

The histological investigation of liver tissues, proved the manifestation of damage caused by cisplatin in comparison to the normal control group and the improvement of tissue structure upon the administration of *C. annua* extract as a prophylaxis before cisplatin injection. Figure 1A1 represents a photomicrograph of the normal control group which received the vehicle only, where no remarkable deviation from the normal histological architecture of hepatocytes was noticed. On the contrary, cisplatin group exhibited prominent signs of liver injury particularly in the portal area where hydropic degeneration of hepatocytes, congestion, dilatation of sinusoids, infiltration of inflammatory cells and focal necrosis were observed compared to normal control group (Figure 1A2). However, pretreatment of the experimental animals with *C. annua* extract before cisplatin injection has noticeably ameliorated cisplatin inflicted liver damage, where the degeneration and necrosis of the hepatocytes were diminished as well as congestion and the inflammatory infiltration were reduced compared to cisplatin group (Figure 1A3). 

On the other hand, renal tissues of cisplatin treated rats exhibited prominent kidney damage including glomerular atrophy, inflammatory cell infiltration as well as degeneration, dilatation and necrosis of renal tubules compared to control group which displayed normal renal histological features without apparent abnormalities (Figure 1B2). Administration of *C. annua* extract as a prophylaxis displayed remarkable improvement in histological changes of renal tissues where dilatation, degeneration and necrosis of renal tubules were reduced as well as preservation of glomerular architecture was noticed (Figure 1B3).

#### 3.1.2. Liver Function Tests

The liver enzymes; ALT and AST activities were estimated to inspect the protective effect of *C. annua* extract on liver damage inflicted by cisplatin administration. As shown in Table 1, rats in the cisplatin group exhibited a marked increase in serum levels of AST and ALT enzyme activity in comparison to the normal control group at *p* < 0.05. While treatment with a prophylactic dose of *C. annua* herb remarkably reduced AST and ALT serum levels compared to the cisplatin group.

AST and ALT serum levels are very accurate indicators utilized to diagnose different liver problems. Upon destruction of hepatocytes plasma membrane, these enzymes existing in the cytosol flow into the blood stream. Quantification of serum levels of such enzymes provide insights into the type and extent of liver damage. ALT and AST levels within liver cells are approximately 1000-fold greater than that of the serum. Hence, the serum concentration of these enzymes will be doubled if necrosis occurs in 1% of hepatocytes. Since ALT is present mainly in hepatocytes’ cytosol and mitochondria, ALT is one of the most precise biochemical markers for evaluation of liver functions [55].

Hepatotoxicity induced by cisplatin was noticed in this study through the drastic elevation in serum activities of AST and ALT. This was in agreement with studies reported in [56,57]. However, this was reversed upon the administration of *C. annua* prophylactic dose which led to significant decrease levels of AST and ALT. Therefore, these results evinced that *C. annua* has ameliorated cisplatin induced liver dysfunction.

#### 3.1.3. Kidney Function Tests

Serum creatinine levels and blood urea nitrogen (BUN) were checked to pursue the possible role of *C. annua* in alleviating the deteriorated kidney function caused by Cisplatin treatment. As illustrated in Table 1 cisplatin group showed a substantial rise in both serum creatinine and urea concentrations compared to the normal control group. While, group 3 which received a prophylactic dose of *C. annua* herb before cisplatin injection showed considerable reduction in both serum creatinine and blood urea nitrogen (BUN) concentrations compared to the cisplatin group.

The impairment of renal function by cisplatin was previously reported by many studies [58,59,60]. Cisplatin induces mesangial cells (specialized cells in the kidney) contraction, modifies the ultrafiltration coefficient factors as well as changes the surface area of filtration. These changes will diminish the glomerular filtration rate (GFR) causing alteration in glomerular functions and hence increasing serum concentrations of creatinine and BUN [61]. In the contrary, the intake of *C. annua* before cisplatin injection has obviously and significantly lowered both serum creatinine and BUN suggesting its role in counteracting cisplatin adverse effects on the kidneys.

#### 3.1.4. Malondialdehyde (MDA) and Reduced Glutathione Concentrations

Cisplatin inflicts oxidative stress the liver and kidneys by triggering the formation of reactive oxygen species (ROS) in their tissues via enhancement of NADPH oxidase, xanthine oxidase, cytochrome P450 enzymes and adenosine deaminase activities [62,63]. As a consequent of the huge amount of ROS released that exceeds the normal detoxification capacity, lipid peroxidation, cellular damage, protein oxidation besides interference with signal pathways (transcription factor activation and angiogenesis) take place [64]. 

The in vitro antioxidant potential of *C. annua* was reported in our previous work [36]. Therefore, it was reasonable to verify such activity in vivo through the estimation of tissue MDA and GSH levels which in turn gives insights in its power in reversing cisplatin sparked oxidative stress.

As seen in Table 2 cisplatin group displayed significant rise in MDA levels along with remarkable decrease in reduced-GSH levels in both liver and kidney tissues as a result of increased lipid peroxidation in these organs compared to the normal control group. These observations were in agreement with [65]. Meanwhile, *C. annua* group exhibited noticeable reduction in MDA levels and observable elevation in reduced-GSH levels in both liver and kidney tissues on comparison with the cisplatin group. Hence, we suggest that *C. annua* could play a protective role against chemotherapy induced oxidative stress and prevent its consequent fetal adverse effects.

#### 3.1.5. Inflammatory Markers (Interferon Gamma and IL-1β) and Apoptotic Marker (Caspase 3)

Inflammation participates in cisplatin induced toxicities [66]. Since cisplatin cause a myriad of inflammatory cytokines such as interferon gamma (INFγ), and IL-1β [67]. INFγ is involved in the activation of signal transduction pathways of both innate and acquired immune system [68]. Interleukin-1β (IL-1β) unleashes inflammatory mediators and stimulates chemotaxis of neutrophils and other inflammatory cells into the lesion. Consequently, a cascade of inflammation and tissue injury occur [67].

Additionally, apoptosis is triggered by cisplatin through increasing the pro-apoptotic gene BAX expression and decreasing the anti-apoptotic gene Bcl-2 expression [69,70,71,72]. As a result of BAX binding to the mitochondrial membrane cytochrome C is released in the cytoplasm. As a consequence, activation of caspase Smac and Omi takes place. Both of them stimulate the initiator procaspase-9, which in turn energizes caspase-9 and then caspase- 3 that interacts with various protein substrates, prompting apoptosis.

Therefore, in the present study we detected INFγ and caspase 3 levels to investigate the ability of *C. annua* herb to oppose the inflammation and apoptosis cause by cisplatin injection.

**Figure 2:**
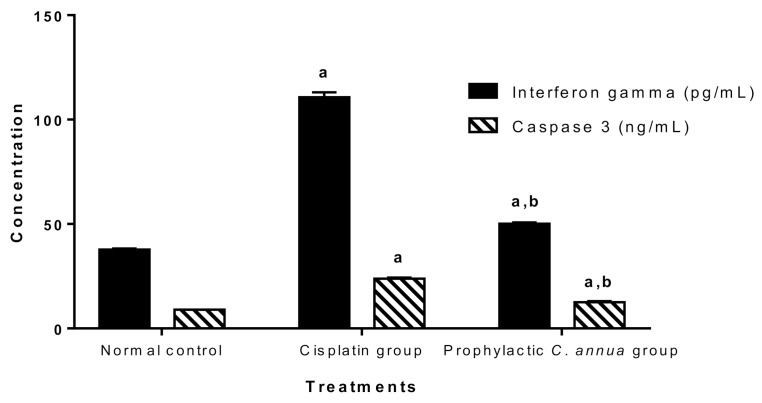
Inflammatory marker (interferon gamma) and apoptotic marker (caspase 3) in the studied groups. Data are represented as mean ± SE and analyzed using ANOVA followed by Bonferroni’s post hoc test at *p* value < 0.05, *n* = 10 for each group. ^a^ indicates the presence of significant difference from normal control group, ^b^ indicates the presence of significant difference from cisplatin group.

Figure 2 demonstrated that cisplatin group had a significant higher level of both INFγ and caspase 3 (110.71 pg/mL, 23.82 ng/mL) respectively compared to the normal control group (37.73 pg/mL, 8.95 ng/mL) respectively at *p* < 0.05. On the other hand, pretreatment of group 3 with *C. annua* led to a significant reduction in the levels of interferon gamma and caspase 3 (50.17 pg/mL, 12.57 ng/mL) respectively in comparison with corresponding cisplatin treated group.

As expected, and in accordance with the literature [73,74,75], the cisplatin group recorded an extreme elevation in INFγ and caspase 3 serum levels indicating massive inflammation and apoptosis, whereas group 3 which received *C. annua* as a prophylactic treatment coped with cisplatin-sparked inflammation and apoptosis as concluded from the noticeable decrease in INFγ and caspase 3 concentrations.

**Figure 3:**
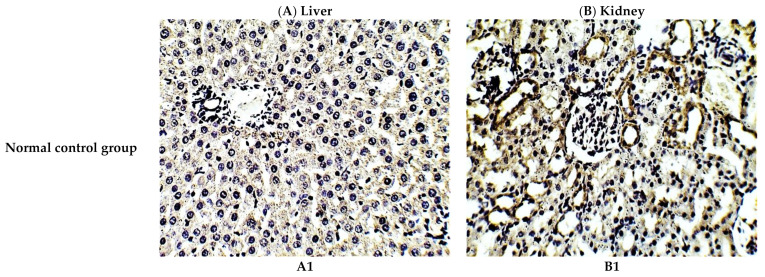

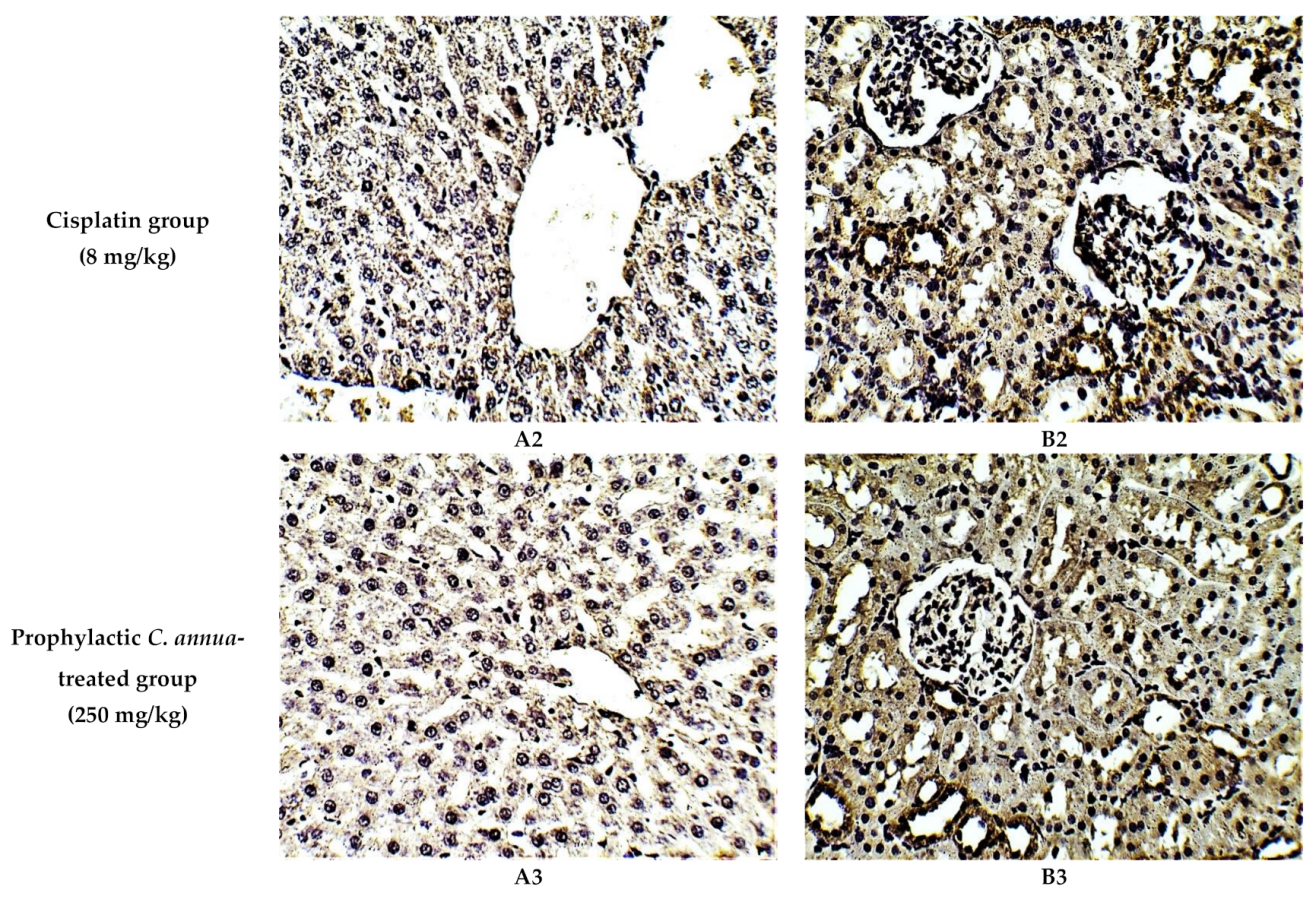
Representative photomicrographs (×400) of immuno-histochemical staining of IL-1β in liver (**A**) and kidney (**B**) tissues of the experimental groups. (**A1**) liver tissues of normal control group, (**A2**) liver tissues of cisplatin group, (**A3**) liver tissues of Prophylactic *C. annua*- treated group, (**B1**) kidney tissues of normal control group, (**B2**) kidney tissues of cisplatin group, (**B3**) kidney tissues of Prophylactic *C. annua*- treated group.

**Figure 4:**
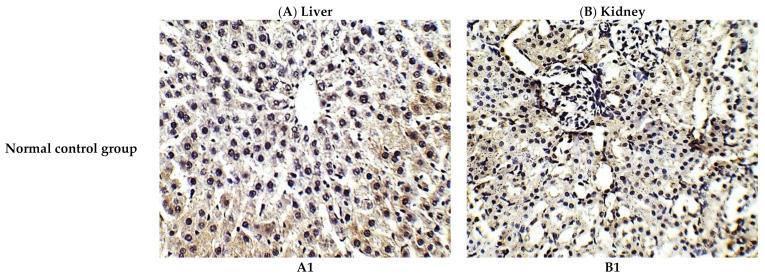

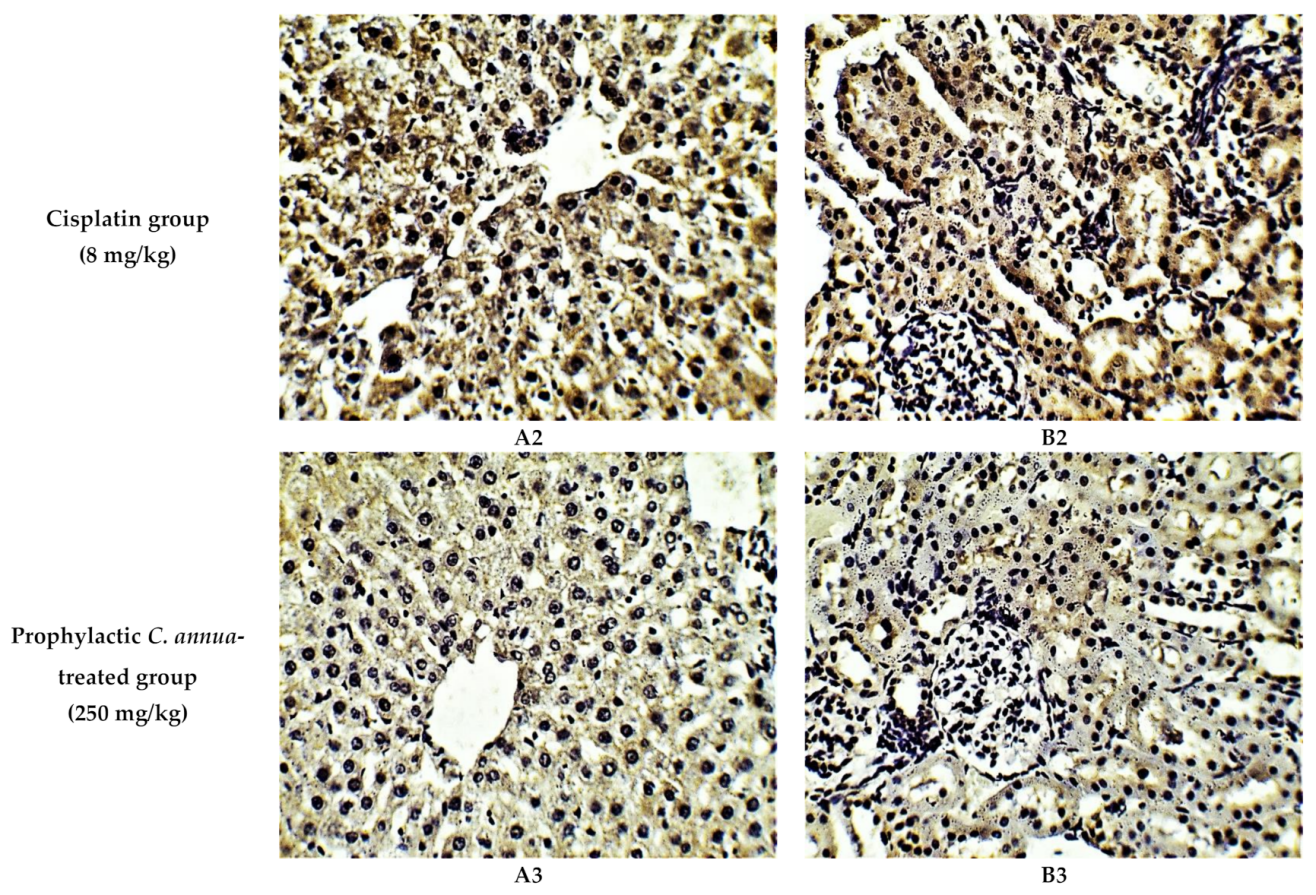
Representative photomicrographs (×400) of immuno-histochemical staining of caspase-3 in liver (**A**) and kidney (**B**) tissues of the experimental groups. (**A1**) liver tissues of normal control group, (**A2**) liver tissues of cisplatin group, (**A3**) liver tissues of Prophylactic *C. annua*- treated group, (**B1**) kidney tissues of normal control group, (**B2**) kidney tissues of cisplatin group, (**B3**) kidney tissues of Prophylactic *C. annua*- treated group.

**Figure 5:**
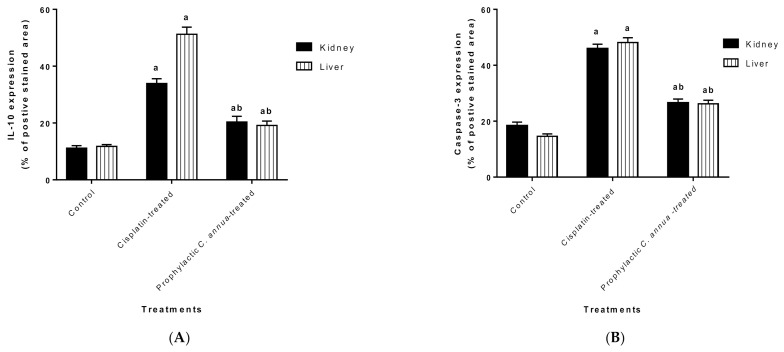
(**A**) Level of IL-1β expression in liver and kidney tissues of the experimental groups. Positive proportions of IL-1β expression were increased significantly in liver and kidney of cisplatin treated rats compared to the normal control and *C. annua* pretreated group. (**B**) Level of caspase-3 expression in liver and kidney tissues of the experimental groups. Positive proportions of caspase-3 expression were elevated remarkably in liver and kidney of cisplatin treated rats compared to the normal control and *C. annua* pretreated group. Data are represented as mean ± SE and analyzed using ANOVA followed by Bonferroni’s post-hoc test at *p* value < 0.05, *n* = 15 for each group. ^a^ indicates the presence of significant difference from normal control group, ^b^ indicates the presence of significant difference from cisplatin group.

The protective effect of *C. annua* extract against cisplatin sparked inflammation and apoptosis in renal and hepatic tissues was further confirmed by immunohistochemistry (IHC) where the expression of both IL-1β (an inflammation marker) and caspase-3 (an apoptosis marker) were investigated. Cisplatin group exhibited pronounced expression of IL-1β and caspase-3 in both renal and hepatic tissues of cisplatin treated group compared to the normal control group. Pretreatment with *C. annua* extract before cisplatin injection has noticeably reduced IL-1β and caspase-3 expression in both kidney and liver tissues in comparison with the cisplatin group (Figure 3, Figure 4, Figure 5).

#### 3.1.6. Mitochondrial DNA (mtDNA) Quantity

Infliction of massive mitochondrial injury is among the mechanisms by which cisplatin prompts its hepatotoxicity and nephrotoxicity leading to mitochondrial DNA depletion and consequently cell death [13,14]. Inside the cells, cisplatin is bio-transformed into a positively charged compound which in turn aggregates inside the mitochondria that are negatively charged organelles targeting its components. Lots of evidence supports the notion that cisplatin cytotoxicity is mediated by its interaction with both nuclear (nDNA) and mitochondrial DNA producing intra-strand and inter-strand DNA adducts. However, mtDNA is more vulnerable to cisplatin induced DNA compare to nDNA due to its lack of histones and efficient DNA repair mechanisms [76].

The mtDNA was quantified in the current study to detect the extent to which *C. annua* intake has reversed mitochondrial damage in liver and kidney cells caused by cisplatin injection.

**Figure 6:**
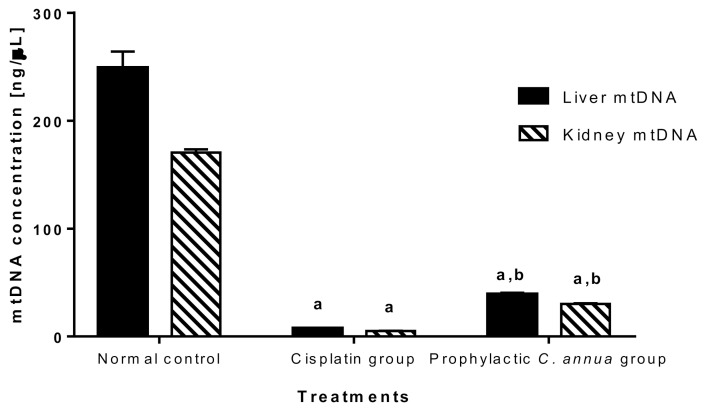
Mitochondrial DNA concentration in liver and kidney tissues in the studied groups. Data are represented as mean ± SE and analyzed using ANOVA followed by Bonferroni’s post-hoc test at *p* value < 0.05, *n* = 10 for each group. ^a^ indicates the presence of significant difference from normal control group, ^b^ indicates the presence of significant difference from cisplatin group.

As shown in Figure 6, mitochondrial DNA was vastly depleted in cisplatin group (concentration = 7.43 ng/μL in liver, concentration = 4.99 ng/μL in kidney) compared to normal control group (concentration = 249 ng/μL in liver, concentration = 170.49 ng/μL in kidney). Group 3 which received a prophylactic dose of *C. annua* herb exhibited a slight but significant increase in mtDNA concentration (39.15 ng/μL in liver, 30.26 ng/μL in kidney) in comparison to cisplatin group at *p* < 0.05. Based on the above observations, we suggested that *C. annua* played a minor role in reversing cisplatin induced mtDNA depletion. Hence, the plant exerted its reno- and hepato-protective activity against cisplatin toxicity by different mechanisms rather than suppression of mitochondrial damage.

### 3.2. In Vitro Anticancer Activity of C. annua—Cisplatin Combination

In the present study, *C. annua* exhibited chemoprotective effect on renal and hepatic tissues by reducing the oxidative stress, inflammation and apoptosis triggered by cisplatin administration. However, cisplatin exerts its anticancer effect via the induction of oxidative stress resulted from the overproduction of reactive oxygen species (ROS) which in turn inflict DNA damage and apoptosis as a consequence [77]. Hence, the assessment of the cytotoxicity of *C. annua* extract–cisplatin combination is mandatory to evaluate whether the co-administration of *C. annua* as adjuvant has affected the chemotherapeutic efficacy of cisplatin or not. In the current investigation, the cytotoxicity of cisplatin individually and in combination with *C. annua* extract was evaluated against the C applying MTT assay. The selection of MCF-7 cell line rather than liver or kidney carcinoma cell lines, was based on our previous study in which we estimated the cytotoxicity of *C. annua* extract against a panel of cancer cell lines. The obtained results revealed that the highest cytotoxic effect of the extract was observed on MCF-7 cell line (IC_50_ = 22.8 µg/mL) [36]. Besides, cisplatin is used in combination with other chemotherapeutic agents for the treatment of breast cancer; metastatic type in particular [78,79]. Moreover, there has been a growing focus on cisplatin-based treatment in recent years for BReast CAncer gene (BRCA)-deficient TNBCs which is marked by poor clinical outcome and represents about 15% to 20% of the recorded cases of breast carcinoma [80,81].

As depicted in Table 3 and supplementary material Figure S19, *C. annua*-cisplatin combination exhibited potent IC50 value of 1.94 μg/mL, compared cisplatin as a single treatment (IC50 = 6.4 μg/mL). These observations evidenced the synergistic effect of the combination which could be explained based on the promising antiproliferative activity of *C. annua* extract previously reported against MCF-7 (IC50 = 22.8 μg/mL [36].

These preliminary findings represent a hopeful approach for overcoming the chemoresistance problem and in turn the adverse effects associated with cisplatin chemotherapy among cancer patients particularly those who suffer from the aggressive types of breast cancer; stage 4 and triple negative breast carcinoma. Hence, there is much work to be done, a future detailed study will be conducted to investigate the cellular mechanisms by which *C. annua* extract has chemo sensitized the MCF-7 towards cisplatin treatment.

### 3.3. Phytochemical Investigation

**Figure 7:**
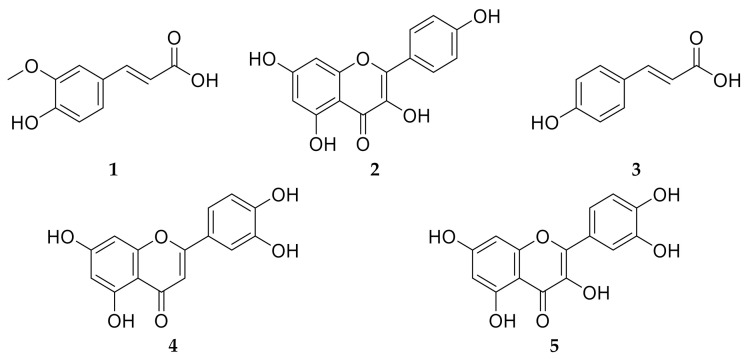
Chemical structures of the isolated compounds **1**–**5** from *C. annua* ethyl acetate fraction. Compound **1** is ferulic acid, compound **2** is kaempferol, compound **3** is *p*-coumaric acid, compound **4** is luteolin and compound **5** is quercetin.

Plants belonging to family Brassicaceae are considered as a rich source of phytoconstituents which account for the reported antioxidant, anticancer, cardiovascular protective and anti-inflammatory activities [82]. *C. annua* have been reported to possess a unique phenolic compound profile [36]. Phytochemical investigation of *C. annua* ethyl acetate fraction has afforded five compounds. Chemical identification of the isolated compounds was achieved by co-chromatography with authentic samples, measuring the melting points and was further confirmed by comparing their ^1^H-NMR and ^13^C-NMR spectral data with the literature. All the NMR spectral data of compounds **1**–**5** were in line with the data published previously. Therefore, the isolated compounds were assigned to be: *trans*-ferulic acid (**1**) [83], kaempferol (**2**) [83,84], *p*-coumaric acid (**3**) [83], luteolin (**4**) [85] and quercetin (**5**) [86] as displayed in Figure 7. It is noteworthy to mention that all the isolated compounds were proved previously to possess in vivo chemoprotective effects against cisplatin reno- and hepato- toxicities [10,27,30,31,32].

### 3.4. Docking Studies

**Figure 8:**
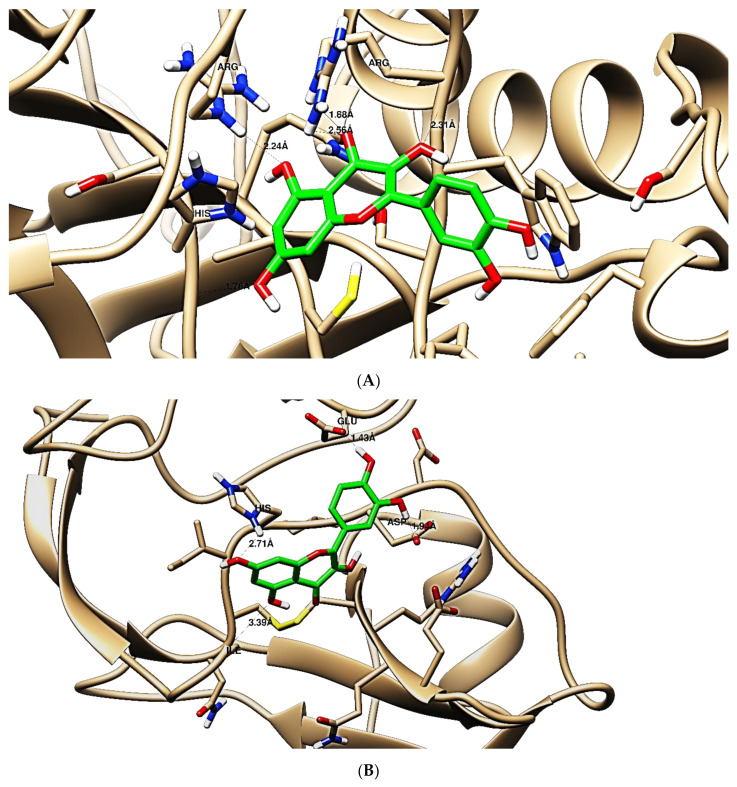
Binding disposition of docked compound **5** inside the active site of caspase-3 (**A**) and IFN γ (**B**) inhibition proteins with interactive key amino acids. Visualization was made using Chimera software [90,91].

The obtained results in this study revealed that *C. annua* extract can successfully counteract cisplatin-triggered hepato- and reno toxicities which is attributed to its phytoconstituents. A phytochemical study of the ethyl acetate fraction of *C. annua* has led to the isolation of five phenolic compounds **1**–**5**: ferulic acid, kaempferol, *p*-coumaric acid, luteolin and quercetin, respectively. The in vivo chemoprotective effects of the isolated compounds against cisplatin induced hepato- and nephrotoxicity were reported in previous studies. Such bioactivities were mediated through the interference with the inflammation and apoptosis processes [10,27,30,31,32]. Moreover, phenolic compounds and flavonoids in particular are known for their anti-inflammatory activity [87,88] besides which they have the ability to reduce apoptosis in normal cells triggered by various stimuli [89]. Therefore, the isolated compounds as well as the other compounds which we identified them previously in *C. annua* extract (mainly phenolics) [29] were scrutinized using protein-ligand docking to predict their binding interactions towards the caspase-3 (PDB:6 CKZ) and IFN-γ (PDB: 2R3Z) proteins obtained from the Protein Data Bank (MOE^®^-2019) in a trial to link these phytochemicals with the experimental results. Table 4 (of the isolated compounds) and Table S6 in the Supplementary Material (of the other identified compounds in *C. annua* extract) show the most favorable poses of the tested compounds and their detailed interactions. All the compounds **1**–**5** isolated in this study as well as most of the previously identified compounds in *C. annua* extract were docked inside the two proteins with good binding interactions: from −12.71 to −20.68 Kcal/mol for caspase-3 protein, and from −13.21 to −15.11 Kcal/mol for IFN-γ protein. They formed good hydrogen bond interactions and lipophilic interactions (arene-cation or arene-arene interactions) with the key interactive amino acids in both active sites of caspase-3 (Arg 207, Arg 64, Gly 122, His 121), and IFN-γ (Ile 12, His 13, Asp 15, Gln 51, and Cys 53). It is noteworthy to mention that the simulation results for the isolated compounds **1**–**5** were in line with the literature as Ekinci Akdemir et al., Bami and coworkers, Sanchez-Gonzalez and his team, the Domitrović research team, and Wang et al. have reported the nephroprotective activity of *p*-coumaric, ferulic acid, quercetin, luteolin and kaempferol, respectively [10,27,30,31,32]. In addition, Ekinci Akdemir and his team demonstrated the hepatoprotection conferred by *p*-coumaric acid [10]. Such bioactivities are exerted via the reduction of apoptosis and inflammation induced by cisplatin. Figure 8 highlighted the three-dimensional binding disposition of the compound (**5**) which exhibited the highest favorable binding energy inside the caspase-3 and IFN-γ inhibitor active sites. The theoretical predictions from the molecular docking study agreed the observed experimental caspase-3 and IFN-γ inhibition activity for the *C. annua* extract suggesting the synergism between its phytoconstituents. 

## 4. Conclusions

In the current study, renal and hepatic toxicities were induced in experimental animals by a single intraperitoneal injection of cisplatin (10 mg/kg). Nevertheless, pretreatment of the rats by *C. annua* extract before cisplatin administration suppressed such toxicities. *C. annua* improved both liver and kidney dysfunctions and ameliorated oxidative stress. Furthermore, it impeded cisplatin induced inflammation and apoptosis and DNA damage. Moreover, the synergism between cisplatin and *C. annua* extract against MCF-7 breast cancer cell was proved. On the other hand, chromatographic study of *C. annua* ethyl acetate fraction yielded five compounds; *trans*-ferulic acid (**1**), kaempferol (**2**), *p*-coumaric acid (**3**), luteolin (**4**) and quercetin (**5**).

The isolated compounds beside the previously identified phytochemicals in *C. annua* extract were inspected for their interaction with caspase-3 and IFN-γ proteins via computational simulations. Interestingly, most of the examined compounds exhibited feasible IFN-γ and caspase-3 inhibition activities demonstrating their anti-inflammatory and anti-apoptotic effects. These observations suggested the anti-nephrotoxic and anti-hepatotoxic potentials of *C. annua* can be attributed to synergism between its phytoconstituents. This study brings a new horizon to a novel therapy to overcome risks of the toxic effects of cisplatin in patients receiving this chemotherapy without affecting its efficacy as a chemotherapeutic drug which in turn may enhance survival and improve the quality of life. Further pharmacological and clinical studies are needed to verify our hypothesis.

## Figures and Tables

**Table 1:** Serum AST, ALT, creatinine and BUN in the studied groups.

Groups	Liver Enzymes		Kidney Parameters	
	AST (U/L)	ALT (U/L)	Creatinine (mg/dL)	BUN (mg/dL)
Normal control group	43.7 ± 0.83	14.5 ± 0.75	0.84 ± 0.018	21.79 ± 0.92
Cisplatin group (8 mg/kg)	124.6 ± 2.16 ^a^	68.5 ± 0.96 ^a^	5.24 ± 0.17 ^a^	210.1 ± 2.6 ^a^
Prophylactic *C. annua*- treated group (250 mg/kg)	69.7 ± 0.97 ^a,b^	32 ± 0.68 ^a,b^	1.81 ± 0.06 ^a,b^	79.78 ± 1.84 ^a,b^

Data are represented as mean ± SE and analyzed using ANOVA followed by Bonferroni’s post-hoc test at *p* value < 0.05, *n* = 10 for each group. ^a^ indicates the presence of significant difference from normal control group, ^b^ indicates the presence of significant difference from cisplatin group. AST; aspartate aminotransferase, ALT; alanine aminotransferase and BUN; blood urea nitrogen.

**Table 2:** Tissue malondialdehyde (MDA) and reduced glutathione concentrations in the studied groups.

Groups	Liver		Kidney	
	MDA (nmol/g tissue)	Reduced-GSH (μg/g tissue)	MDA (nmol/g tissue)	Reduced-GSH (μg/tissue)
Normal control group	24.59 ± 0.18	43.04 ± 0.2	16.03 ± 0.67	39.62 ± 0.13
Cisplatin group (8 mg/kg)	50.86 ± 1.07 ^a^	23.26 ± 0.37 ^a^	34.10 ± 0.12 ^a^	24.54 ± 0.2 ^a^
Prophylactic *C. annua*- treated group (250 mg/kg)	35.48 ± 0.36 ^a,b^	36.88 ± 0.24 ^a,b^	24.39 ± 0.23 ^a,b^	30.12 ± 0.33 ^a,b^

Data are represented as mean ± SE and analyzed using ANOVA followed by Bonferroni’s post-hoc test at *p* value < 0.05, *n* = 10 for each group. ^a^ indicates the presence of significant difference from normal control group, ^b^ indicates the presence of significant difference from cisplatin group.

**Table 3:** IC_50_ values of cisplatin and *C. annua* extract–cisplatin as adjuvant therapy against breast MCF-7 cells using the MTT assay.

Sample	MCF-7 (μg/mL)
Cisplatin	6.4 ± 0.34
*C. annua* extract+ cisplatin	1.94 ± 0.11

Values are expressed as Mean ± SD of triplet trials and calculated using EXCEL using nonlinear regression Dose-Inhibition curve fit.

**Table 4:** Summary of ligand-receptor interactions of the five docked compounds inside the active site of caspase-3 (PDB: 6CKZ) and IFN-γ protein (PDB: 2R3Z).

Compound	Caspase-3 (PDB: 6CKZ) *			IFN-γ (PDB: 2R3Z) ^#^		
1	Arg 207 His 121	2 HB-acceptor and donor 1 HB-acceptor	1 arene-cation with His 121	Ile 12 His 13 Asp 15	1 HB-acceptor 1 HB-acceptor 1 HB-donor	-
	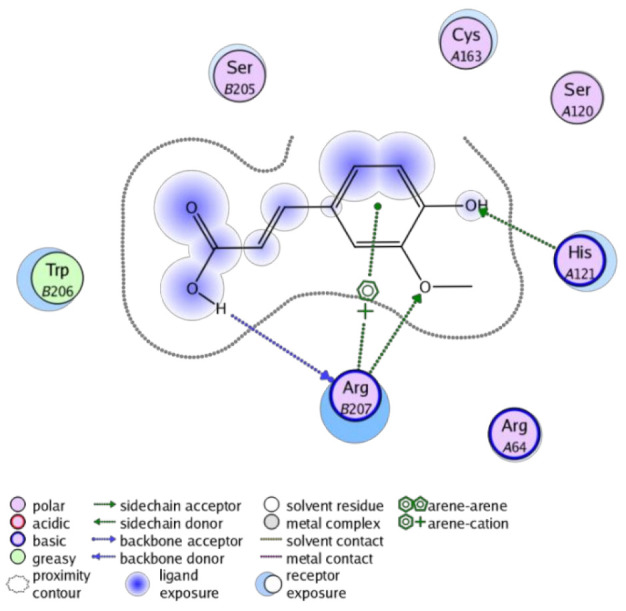			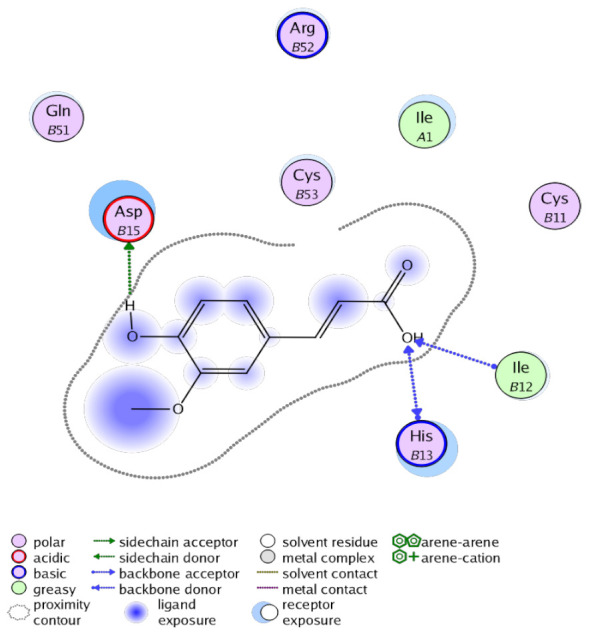		
	Caspase-3 (PDB: 6CKZ) *			**IFN-γ (PDB: 2R3Z) ^#^**		
2	Arg 207 His 121	1 HB-acceptor 1 HB-acceptor	1 arene-cation with Arg 207	Ile 1 His 13 Asp 15 Gln 51	1 HB-acceptor 1 HB-acceptor 1 HB-donor 1 HB-donor	1 arene-cation with Ile 1
	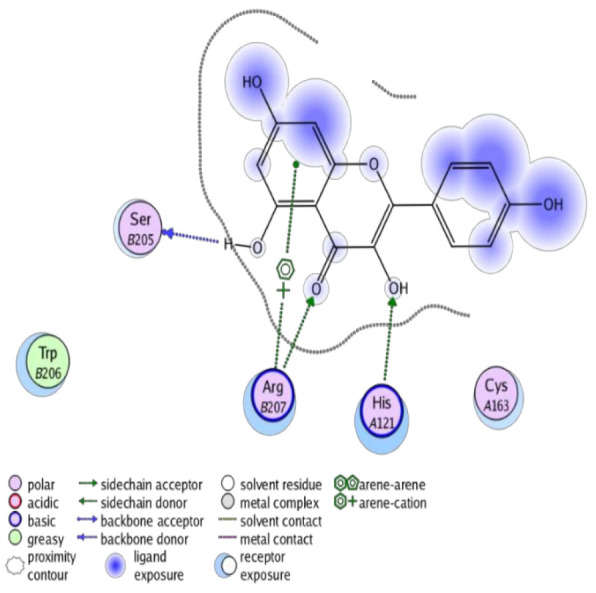			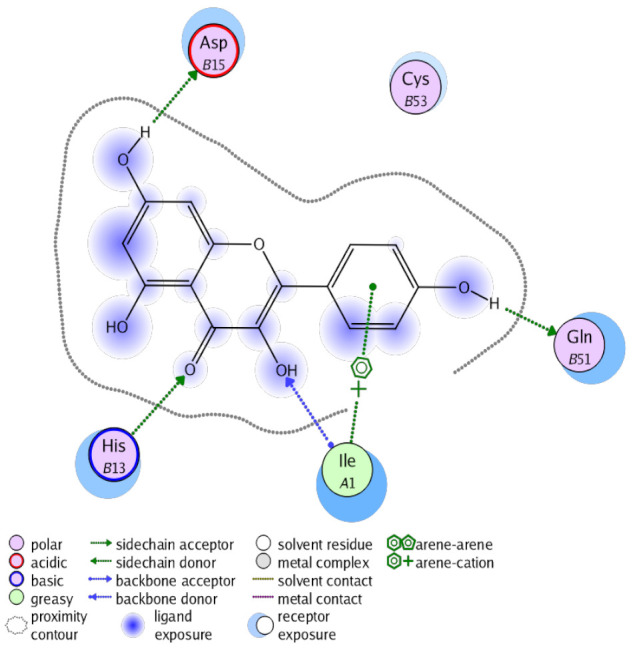		
	Caspase-3 (PDB: 6CKZ) *			**IFN-γ (PDB: 2R3Z) ^#^**		
3	Arg 64	1 HB-acceptor	1 arene-cation with Arg 207	Gln 51 Cys 53	1 HB-donor 1HB-acceptor	1 arene-cation with Ile 1
	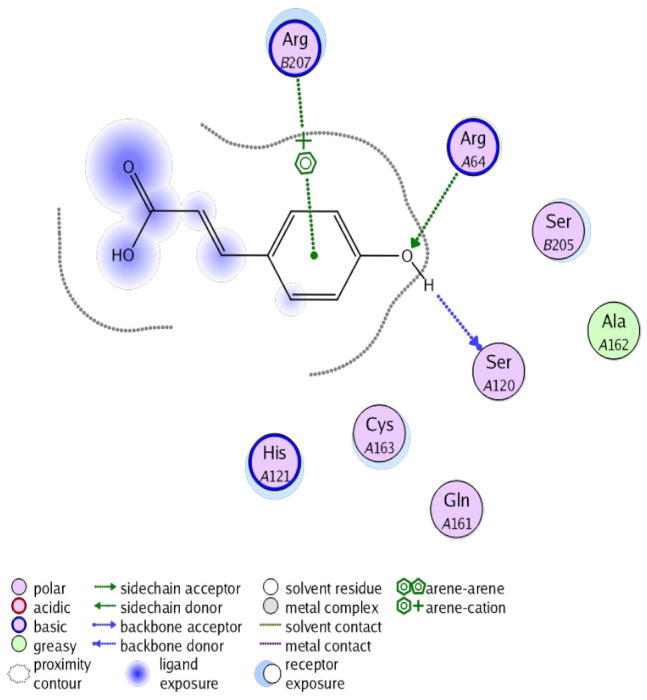			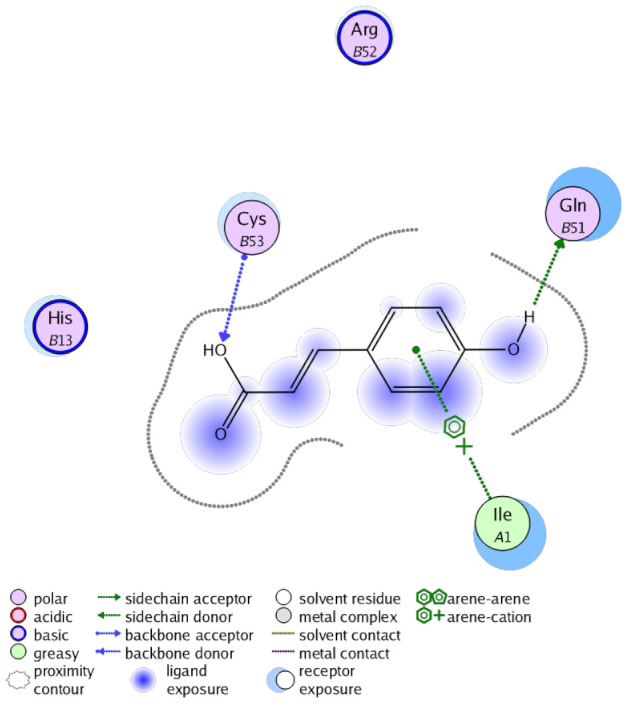		
	Caspase-3 (PDB: 6CKZ) *			**IFN-γ (PDB: 2R3Z) ^#^**		
4	Arg 207 His 121	1 HB-donor I HB-acceptor	1 arene-cation with Arg 207	Ile 1 Cys 53	1 HB-acceptor I HB-acceptor	1 arene-arene with His 13
	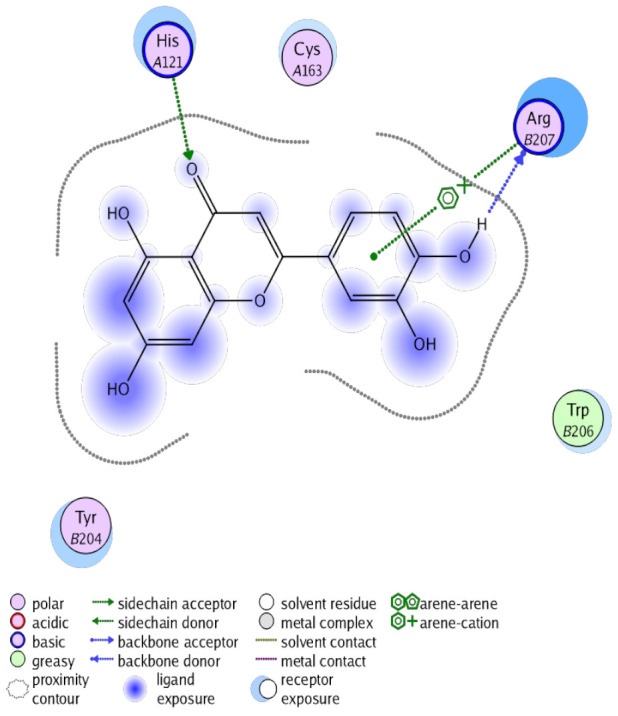			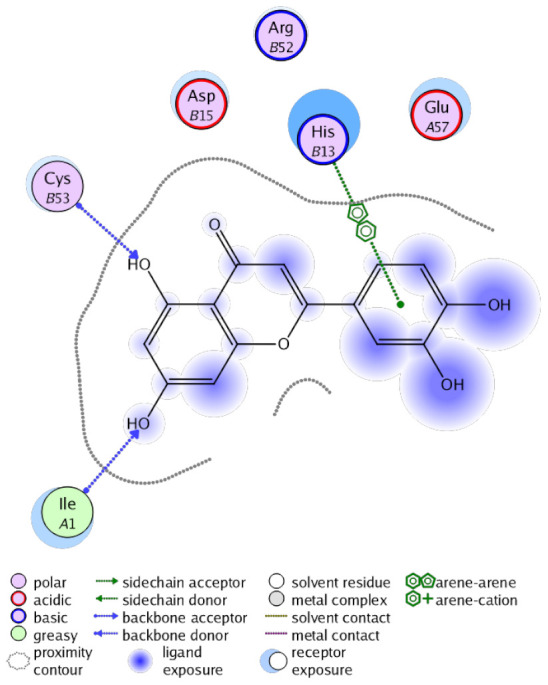		
	Caspase-3 (PDB: 6CKZ) *			**IFN-γ (PDB: 2R3Z) ^#^**		
5	Arg 207 Arg 64 Gly 122	1 HB-acceptor I HB-acceptor I HB-acceptor	1 arene-cation with His 121	Asp 15 Glu 57	1 HB-donor I HB-donor	1 arene-cation with His 13 1 arene-cation with Ile 1
	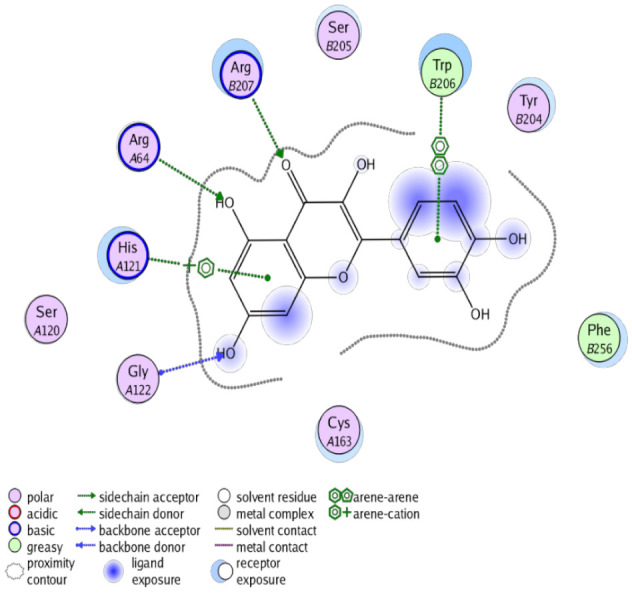			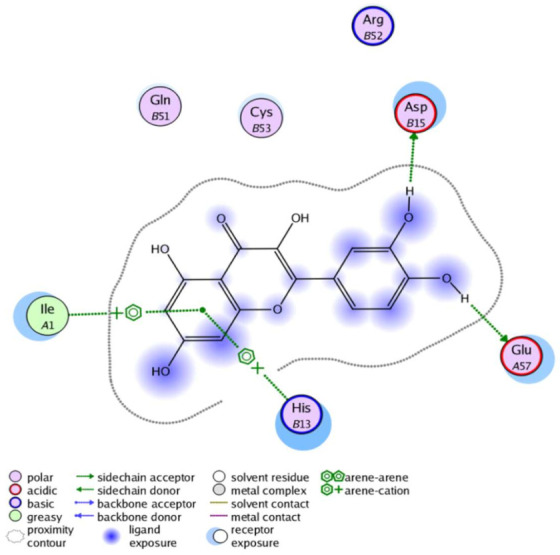		

* The binding energies of the docked compounds inside caspase-3 protein from −12.71 to −20.68 Kcal/mol, ^#^ The binding energies of the docked compounds inside IFN-γ protein from −13.21 to −15.11 Kcal/mol.

## Data Availability

Data is contained within the article and supplementary material.

## Supplementary Materials

The following are available online at https://www.mdpi.com/article/10.3390/antiox10060825/s1, Figures S1–S18, The ^1^H and ^13^C-NMR spectra of compounds **1**–**5**; Figure S19, MTT assay dose-response curve of cisplatin and cisplatin—*C. annua* extract combination on MCF-7 cells; Tables S1–S5: The ^1^H and ^13^C-NMR spectral data as well as the melting point of the isolated compounds **1**–**5**; Table S6, Summary of ligand-receptor interactions of the previously identified compounds in *C. annua* extract towards caspase-3 (PDB = 6 CKZ) and IFN-γ (PDB: 2R3Z).
