# Supplementary material for: The Antioxidant Carrichtera annua DC. Ethanolic Extract Counteracts Cisplatin Triggered Hepatic and Renal Toxicities
Source: Antioxidants (Basel). 2021 May 21;10(6):825. doi: 10.3390/antiox10060825 (PMC8224350; doi:10.3390/antiox10060825)
Supplement: Supplementary file 1 [file antioxidants-10-00825-s001.zip › antioxidants-1198017-supplementary.pdf]

The Antioxidant *Carrichtera annua* DC. Ethanolic Extract Counteracts Cisplatin Triggered Hepatic and Renal Toxicities

Enas E. Eltamany [1,†], Sameh S. Elhady [2,†], Mohamed S. Nafie [3], Haidy A. Ahmed [1,4], Dina M. Abo-Elmatty [5], Safwat A. Ahmed [1], Jihan M. Badr [1,*] and Asmaa R. Abdel-Hamed [5]

[1] Department of Pharmacognosy, Faculty of Pharmacy, Suez Canal University, Ismailia 41522, Egypt; haidyabdelkader@gmail.com (H.A.A.); enastamany@gmail.com (E.E.E.); safwat_ahmed@pharm.suez.edu.eg (S.A.A.); gehan_ibrahim@pharm.suez.edu.eg (J.M.B.)
[2] Department of Natural Products, Faculty of Pharmacy, King Abdulaziz University, Jeddah 21589, Saudi Arabia; ssahmed@kau.edu.sa (S.S.E.)
[3] Department of Chemistry, Faculty of Science, Suez Canal University, Ismailia 41522, Egypt; mohamed_nafie@science.suez.edu.eg (M.S.N.)
[4] Ismailia Health Affairs Directorate, Ismailia 41525, Egypt;
[5] Department of Biochemistry, Faculty of Pharmacy, Suez Canal University, Ismailia 41522, Egypt; dinawahadan@yahoo.com (D.M.A.); asmaa.ramdan@pharm.suez.edu.eg (A.R.A.)
* Correspondence: gehan_ibrahim@pharm.suez.edu.eg; Tel.: +20-010-91332451; Fax: +20-064-3230741
† These authors equally contributed to this work

Additional Experimental Detail

1) **Figure S1.** ^{13}C-NMR (100 MHz, CD_3OD) spectrum of compound **1**
2) **Figure S2.** ^{1}H-NMR (400 MHz, CD_3OD) spectrum of compound **1**
3) **Figure S3.** Partial expansion of ^{1}H-NMR (400 MHz, CD_3OD) spectrum of compound **1**
4) **Figure S4.** Partial expansion of ^{1}H-NMR (400 MHz, CD_3OD) spectrum of compound **1**
5) **Figure S5.** ^{13}C-NMR (100 MHz, CD_3OD) spectrum of compound **2**
6) **Figure S6.** ^{1}H-NMR (400 MHz, CD_3OD) spectrum of compound **2**
7) **Figure S7.** Partial expansion of ^{1}H-NMR (400 MHz, CD_3OD) spectrum of compound **2**
8) **Figure S8.** ^{13}C-NMR (100 MHz, CD_3OD) spectrum of compound **3**
9) **Figure S9.** ^{1}H-NMR (400 MHz, CD_3OD) spectrum of compound **3**
10) **Figure S10.** Partial expansion of ^{1}H-NMR (400 MHz, CD_3OD) spectrum of compound **3**
11) **Figure S11.** ^{13}C-NMR (100 MHz, DMSO-d_6) spectrum of compound **4**
12) **Figure S12.** ^{1}H-NMR (400 MHz, DMSO-d_6) spectrum of compound **4**
13) **Figure S13.** Partial expansion of ^{1}H-NMR (400 MHz, DMSO-d_6) spectrum of compound **4**
14) **Figure S14.** Partial expansion of ^{1}H-NMR (400 MHz, DMSO-d_6) spectrum of compound **4**
15) **Figure S15.** ^{13}C-NMR (100 MHz, DMSO-d_6) spectrum of compound **5**
16) **Figure S16.** ^{1}H-NMR (400 MHz, DMSO-d_6) spectrum of compound **5**
17) **Figure S17.** Partial expansion of ^{1}H-NMR (400 MHz, DMSO-d_6) spectrum of compound **5**
18) **Figure S18.** Partial expansion of ^{1}H-NMR (400 MHz, DMSO-d_6) spectrum of compound **5**
19) **Figure S19.** MTT assay dose-response curve of cisplatin and cisplatin - *C. annua* extract combination on MCF-7 cells
20) **Table S1.** The ^{1}H and ^{13}C-NMR spectral data as well as the melting point of compound **1**
21) **Table S2.** The ^{1}H and ^{13}C-NMR spectral data as well as the melting point of compound **2**

22) **Table S3.** The ^{1}H and ^{13}C-NMR spectral data as well as the melting point of compound **3**
23) **Table S4.** The ^{1}H and ^{13}C-NMR spectral data as well as the melting point of compound **4**
24) **Table S5.** The ^{1}H and ^{13}C-NMR spectral data as well as the melting point of compound **5**
25) **Table S6.** Summary of ligand-receptor interactions of the previously identified compounds in *C. annua* extract towards caspase-3 (PDB=6CKZ) and IFN-γ (PDB: 2R3Z)

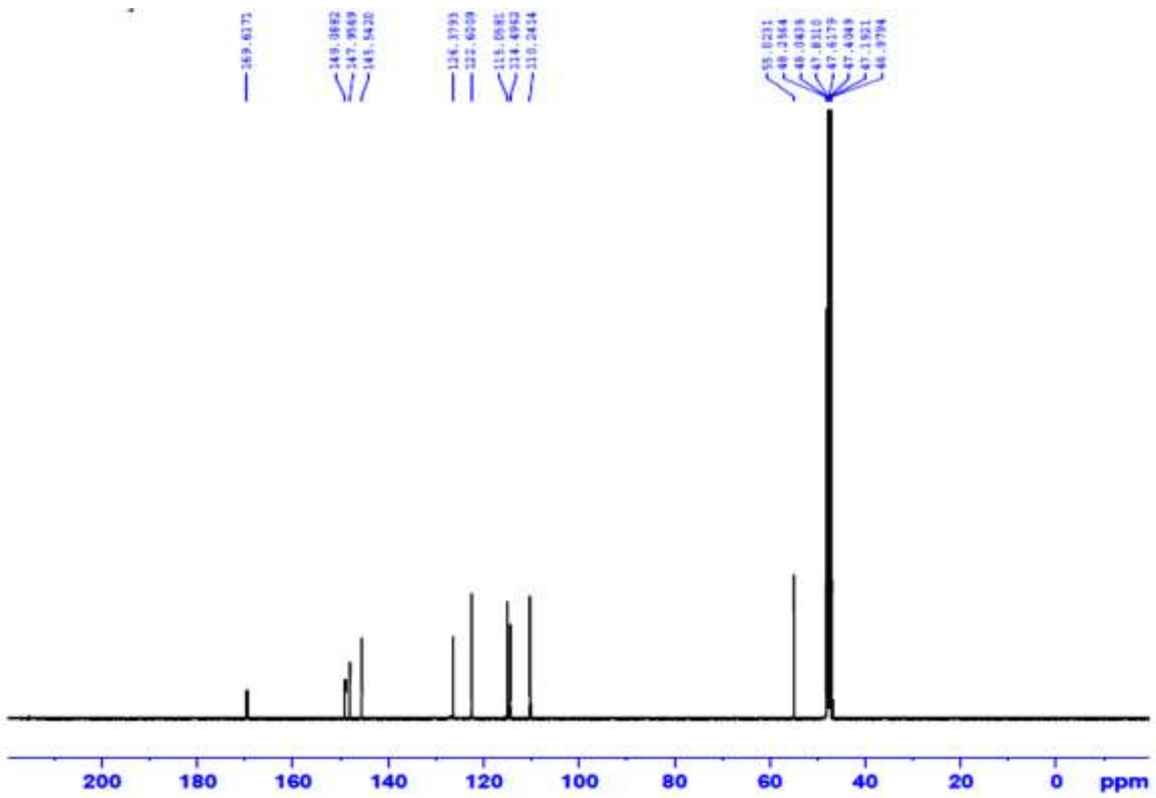

Figure S1. ^{13}C-NMR (100 MHz, CD_3OD) spectrum of compound **1**

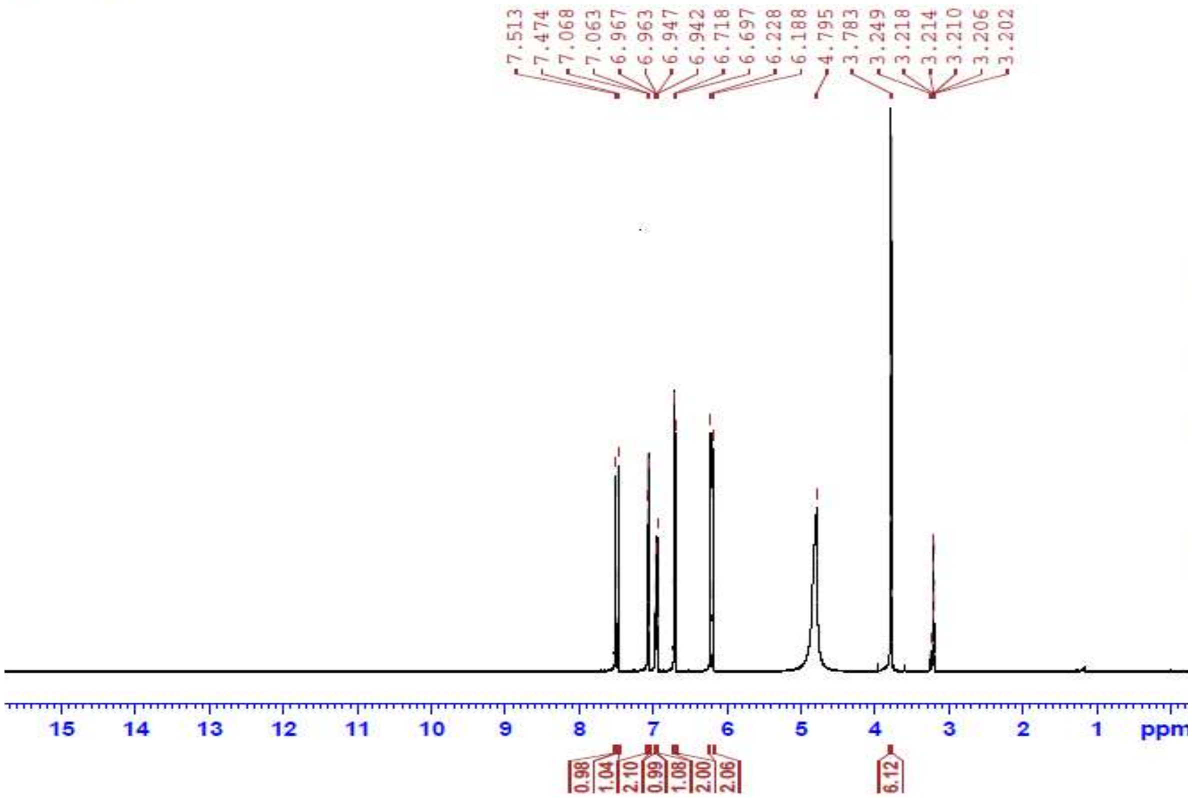

Figure S2. ^{1}H-NMR (400 MHz, CD_3OD) spectrum of the compound **1**

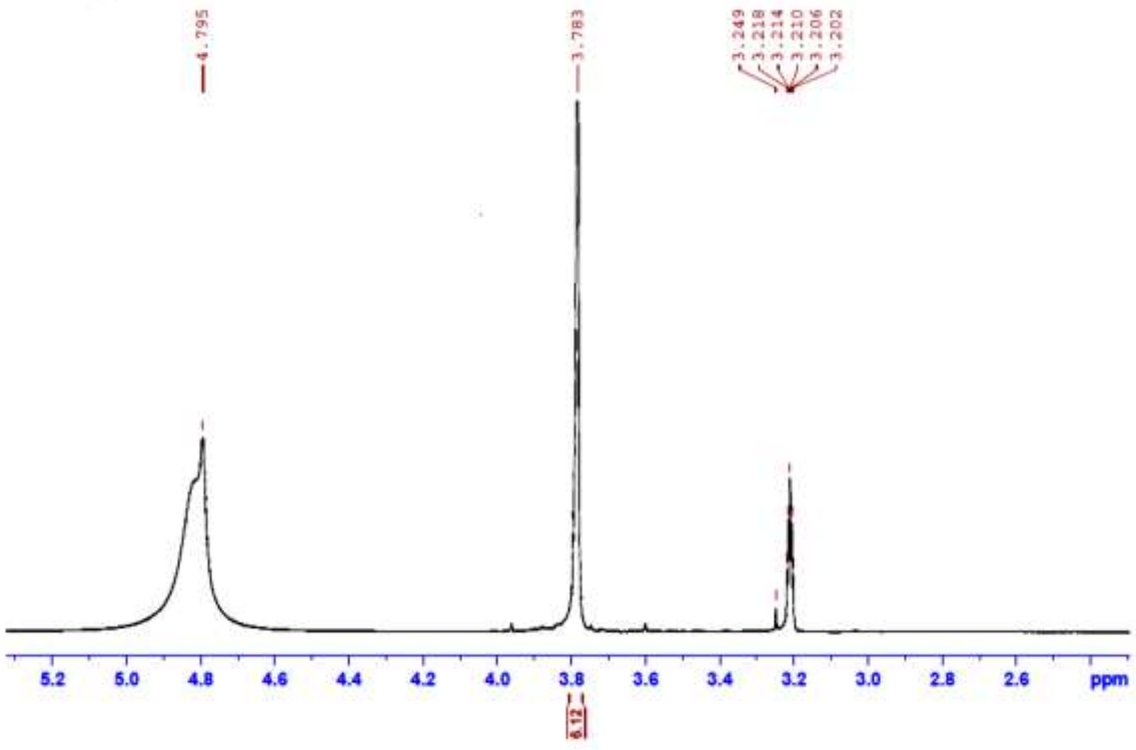

Figure S3. Partial expansion of ^{1}H-NMR (400 MHz, CD_3OD) spectrum of compound **1**

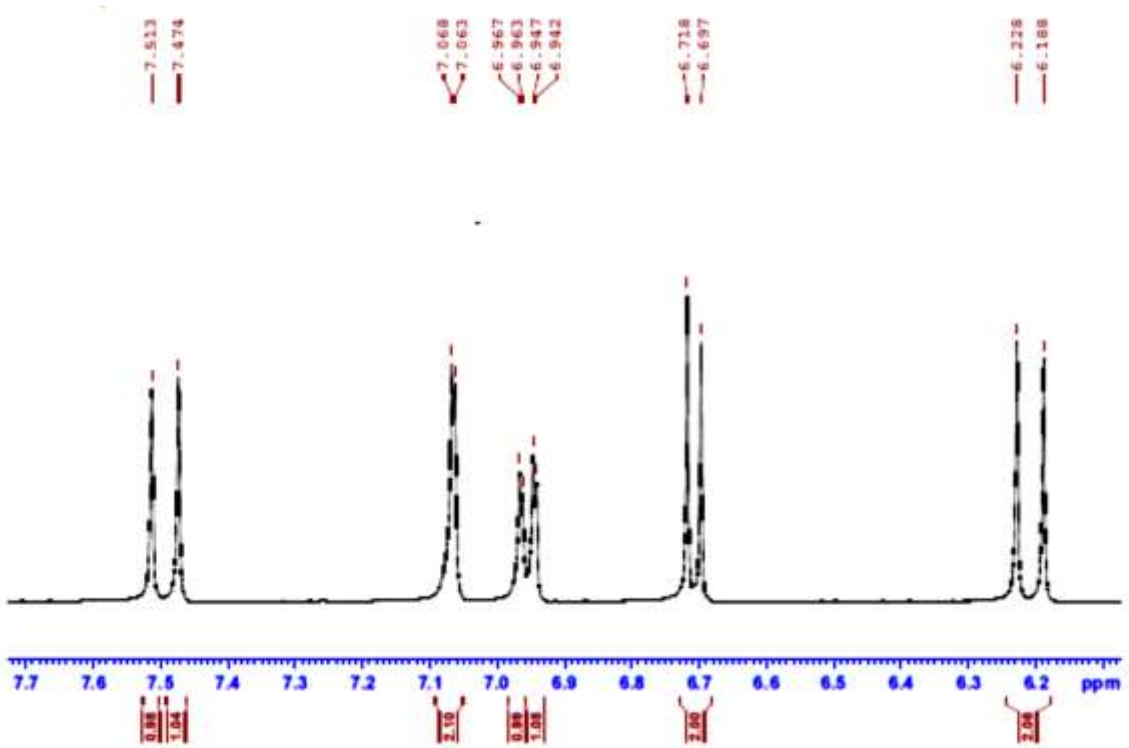

Figure S4. Partial expansion of ^{1}H-NMR (400 MHz, CD_3OD) spectrum of compound **1**

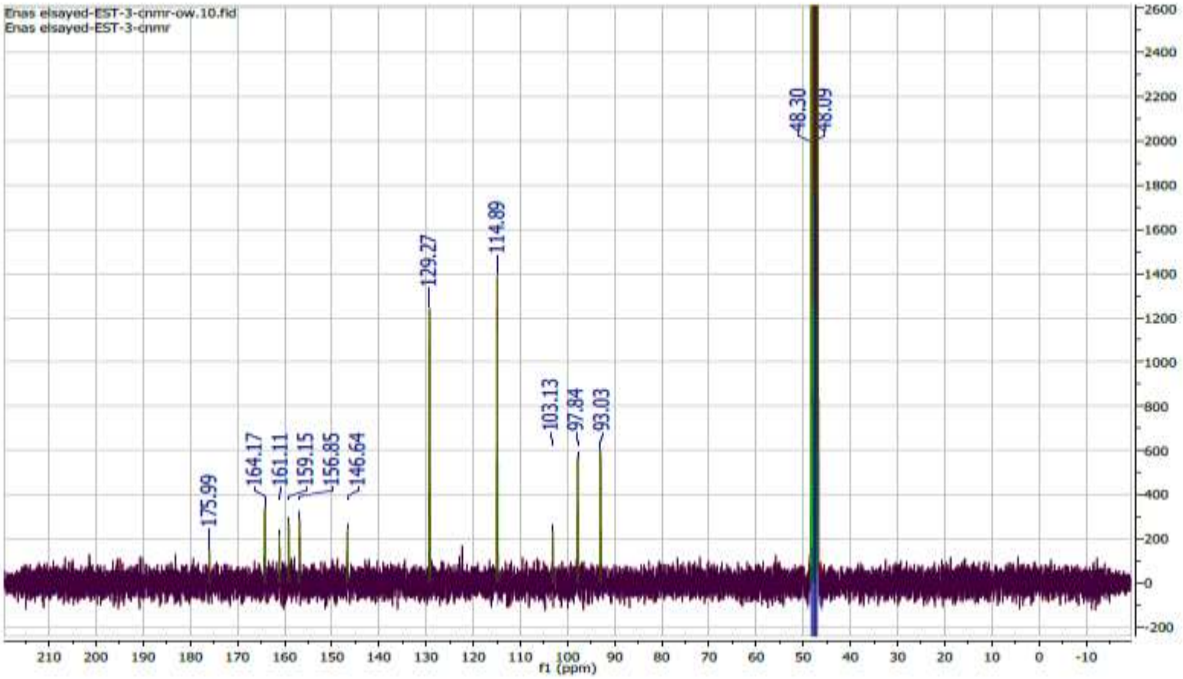

Figure S5. ^{13}C-NMR (100 MHz, CD_3OD) spectrum of compound **2**

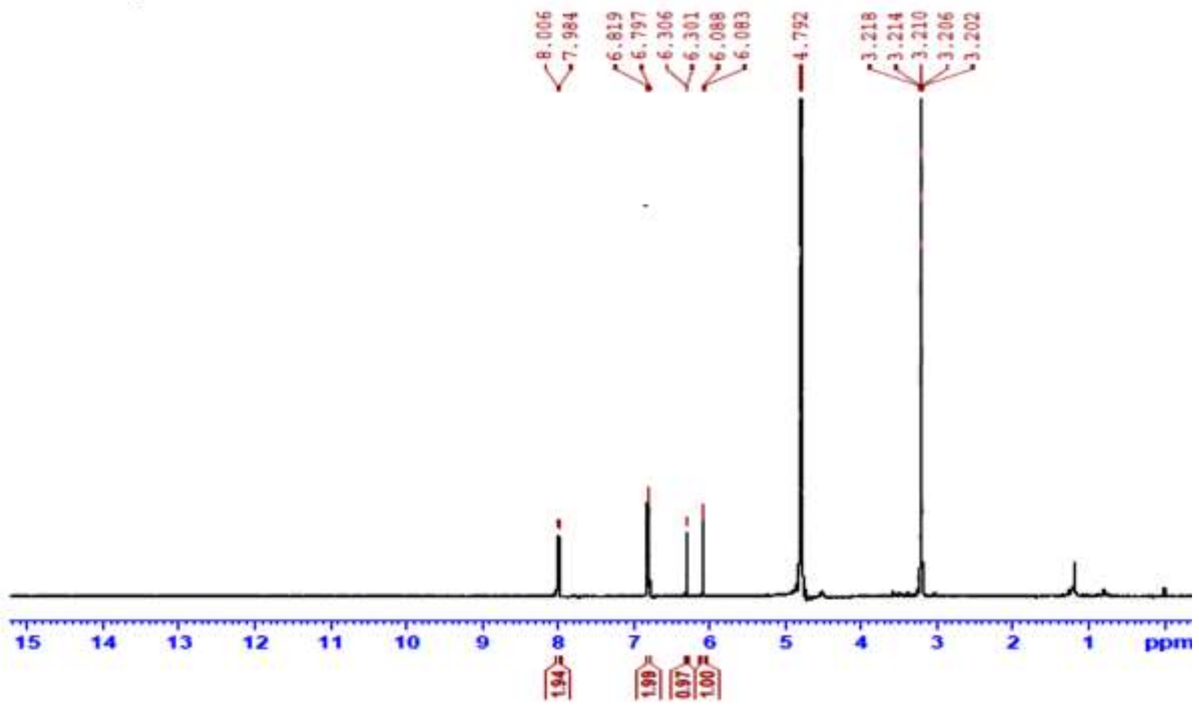

Figure S6. ^{1}H-NMR (400 MHz, CD_3OD) spectrum of compound **2**

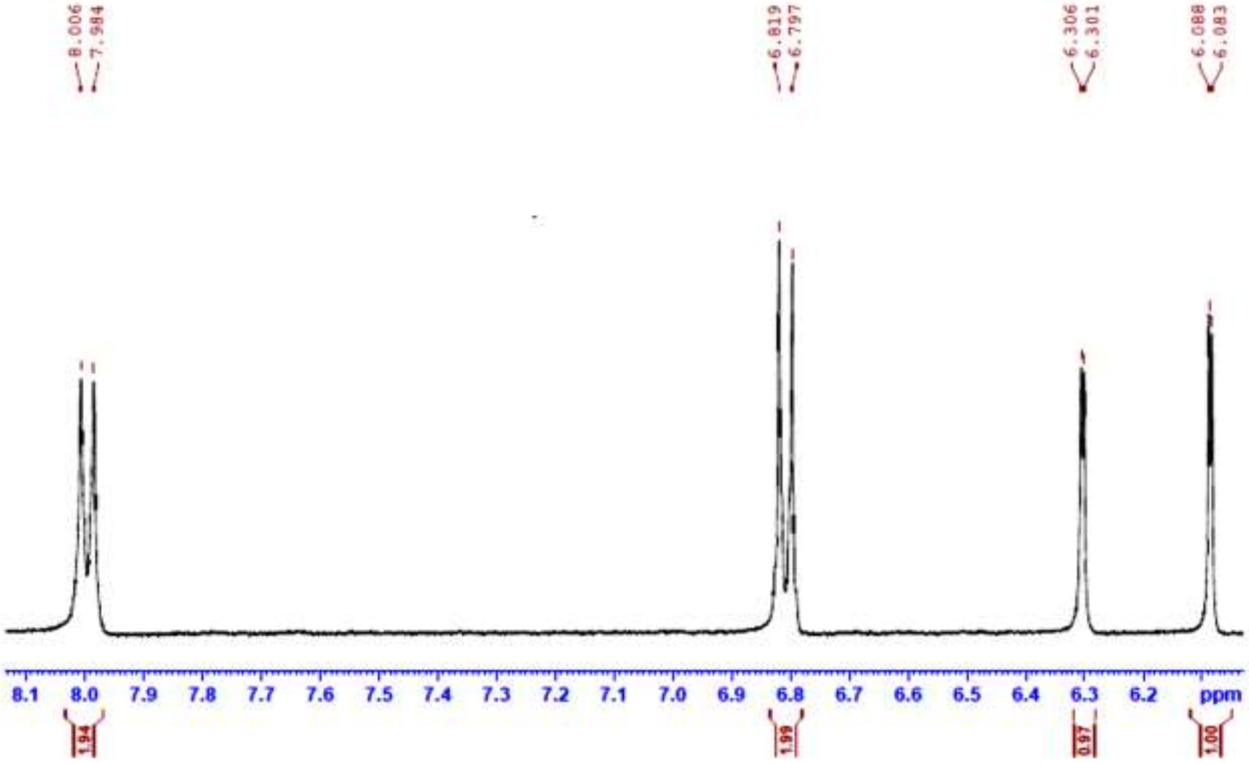

Figure S7. Partial expansion of ^{1}H-NMR (400 MHz, CD_3OD) spectrum of compound **2**

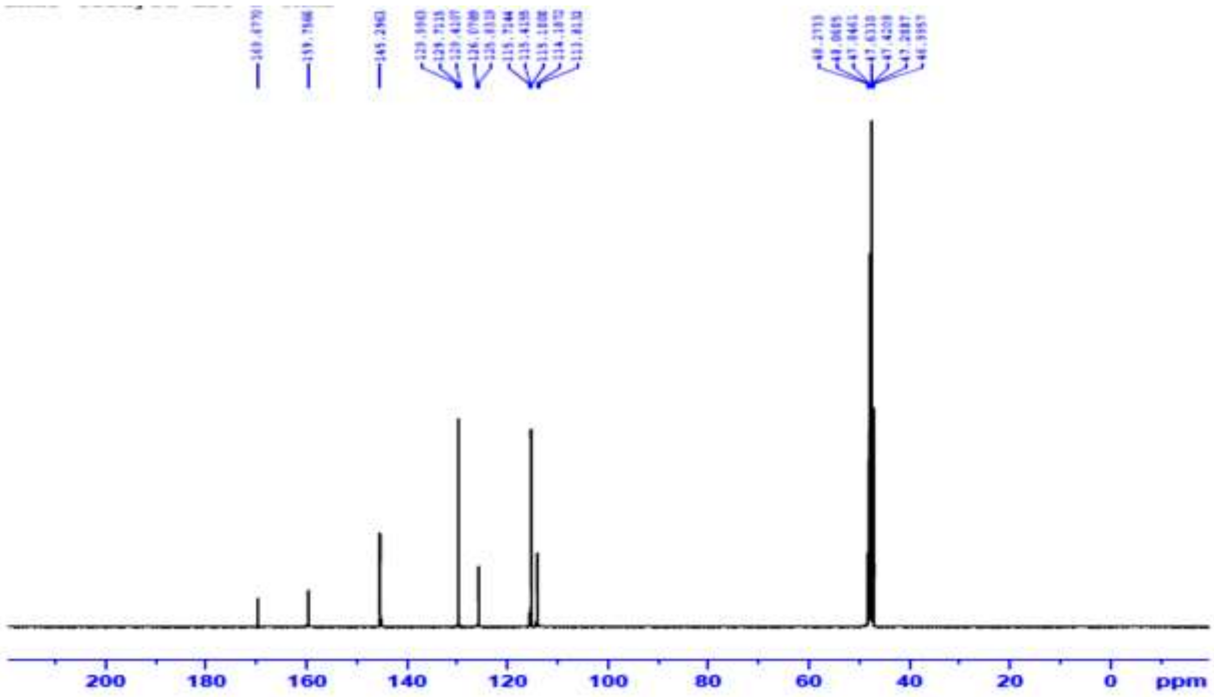

Figure S8. ^{13}C-NMR (100 MHz, CD_3OD) spectrum of compound **3**

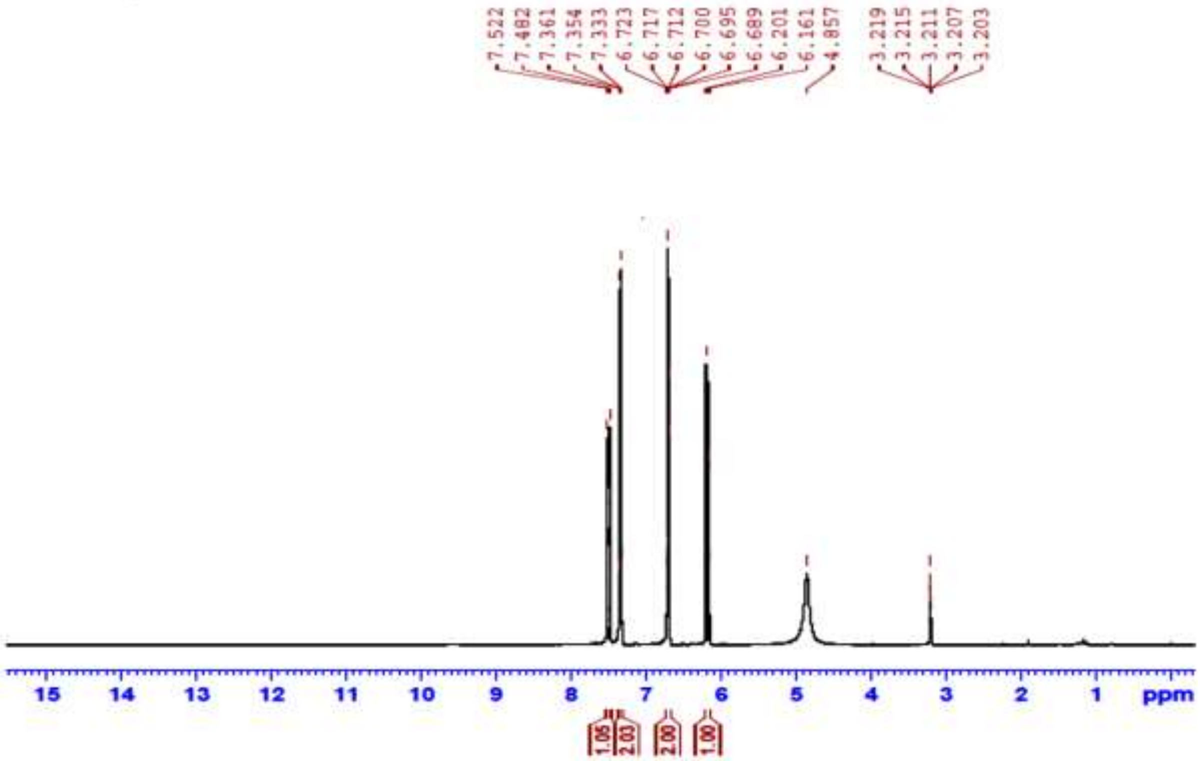

Figure S9. ^{1}H-NMR (400 MHz, CD_3OD) spectrum of compound **3**

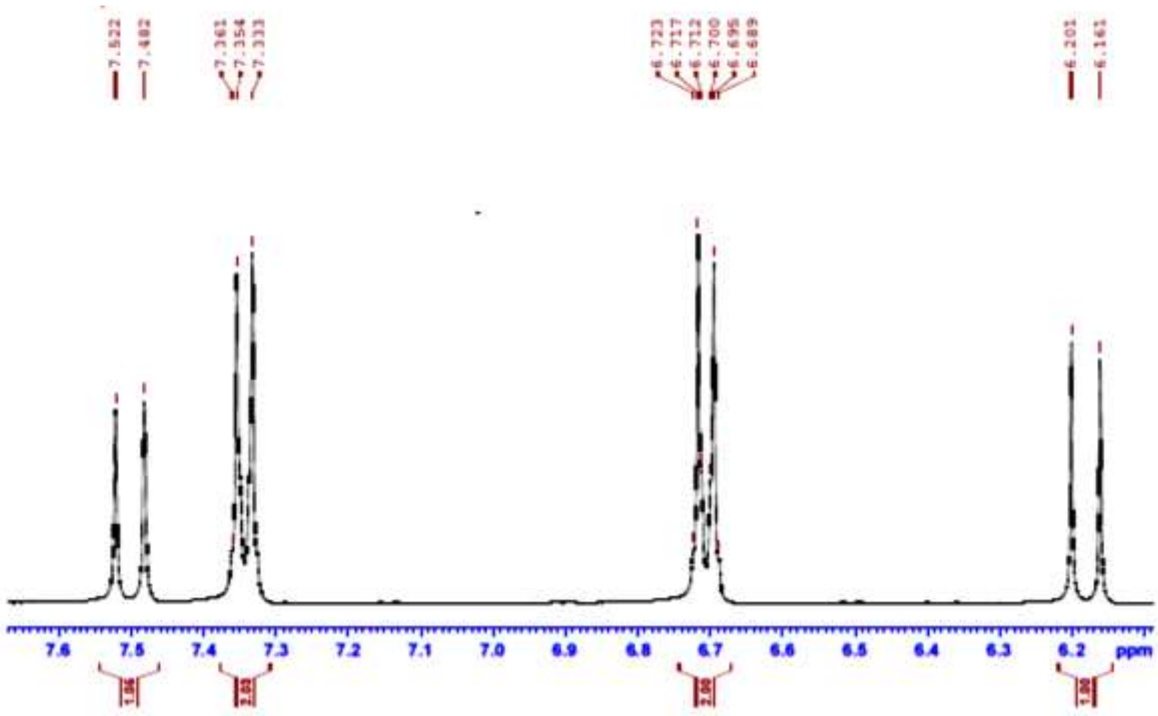

Figure S10. Partial expansion of ^{1}H-NMR (400 MHz, CD_3OD) spectrum of compound **3**

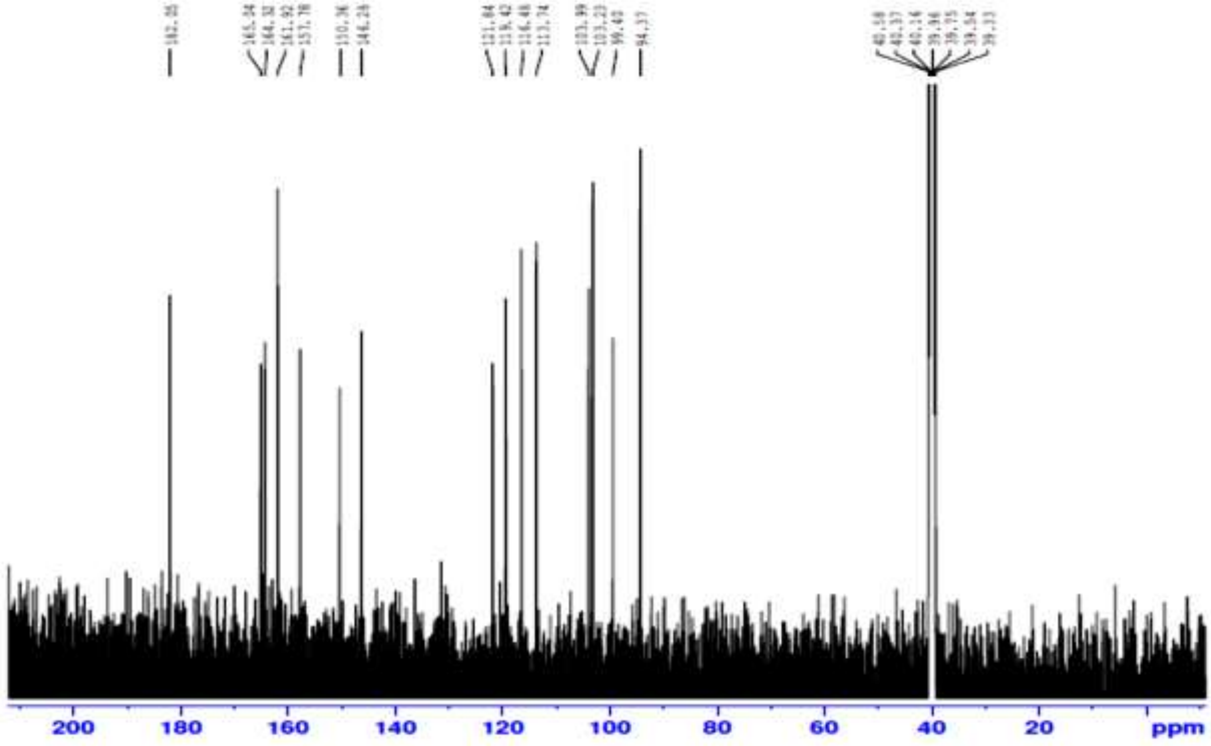

Figure S11. ^{13}C-NMR (100 MHz, DMSO-d_6) spectrum of compound **4**

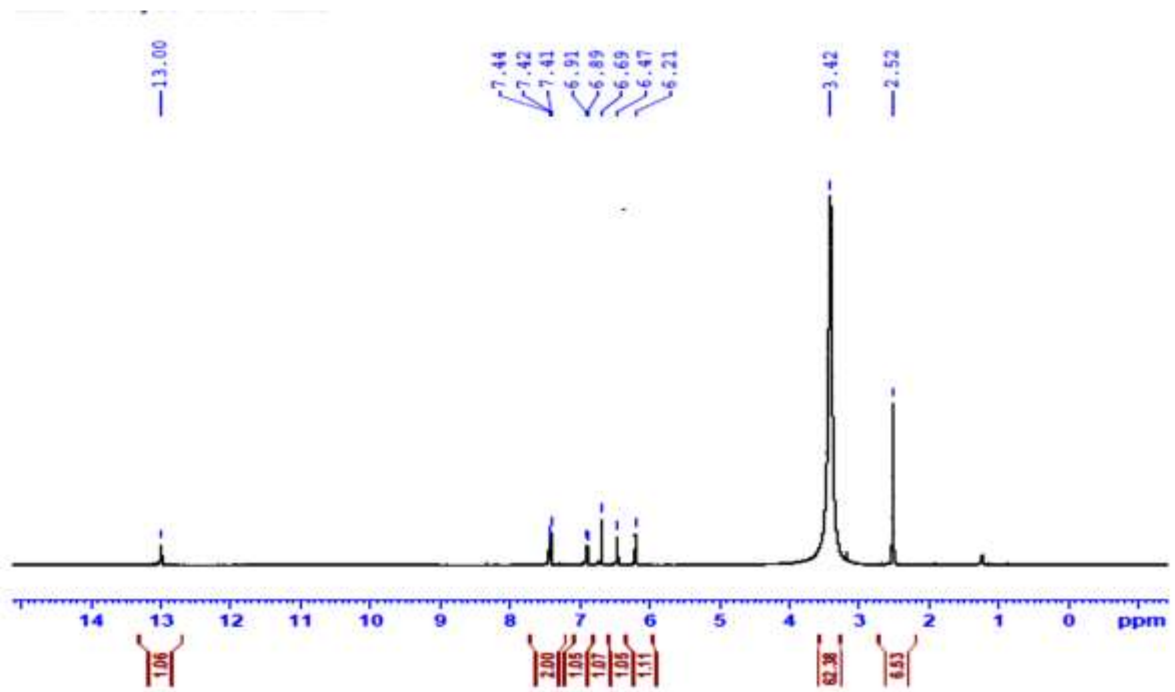

Figure S12. ^{1}H-NMR (400 MHz, DMSO-d_6) spectrum of compound **4**

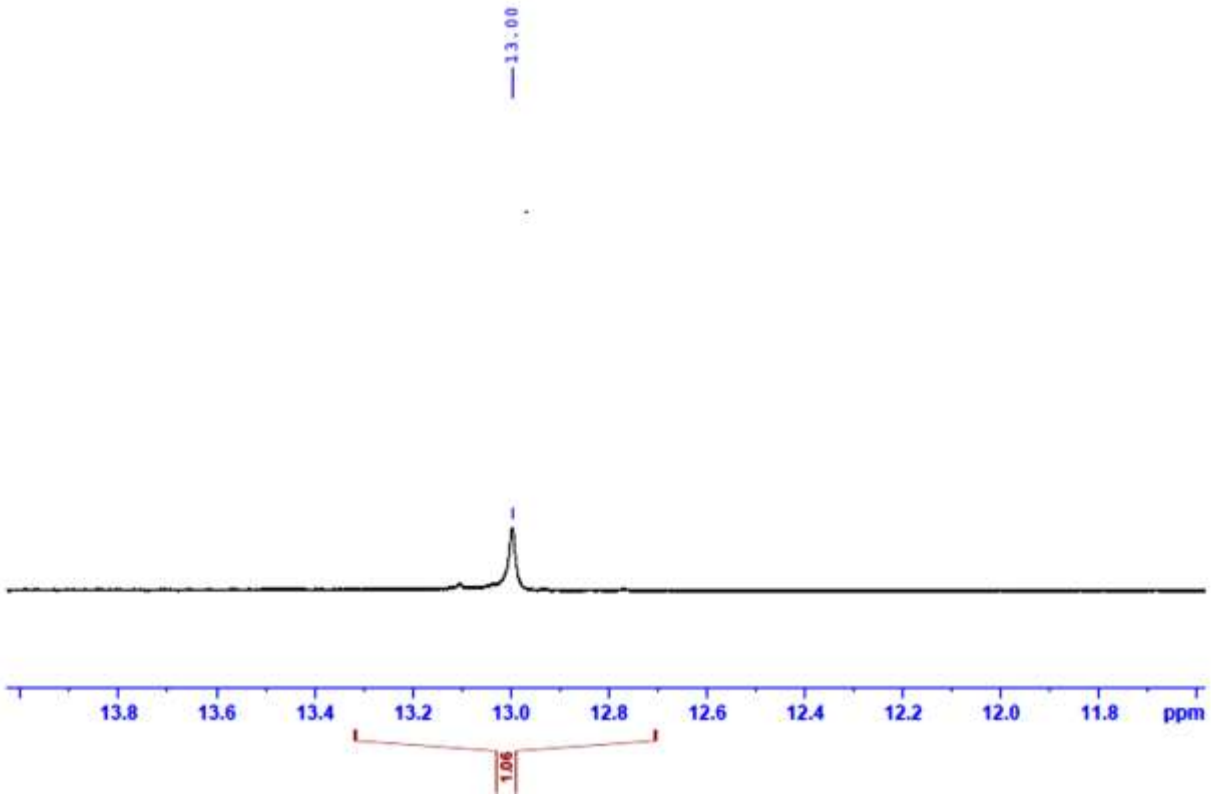

Figure S13. Partial expansion of ^{1}H-NMR (400 MHz, DMSO-d_6) spectrum of compound **4**

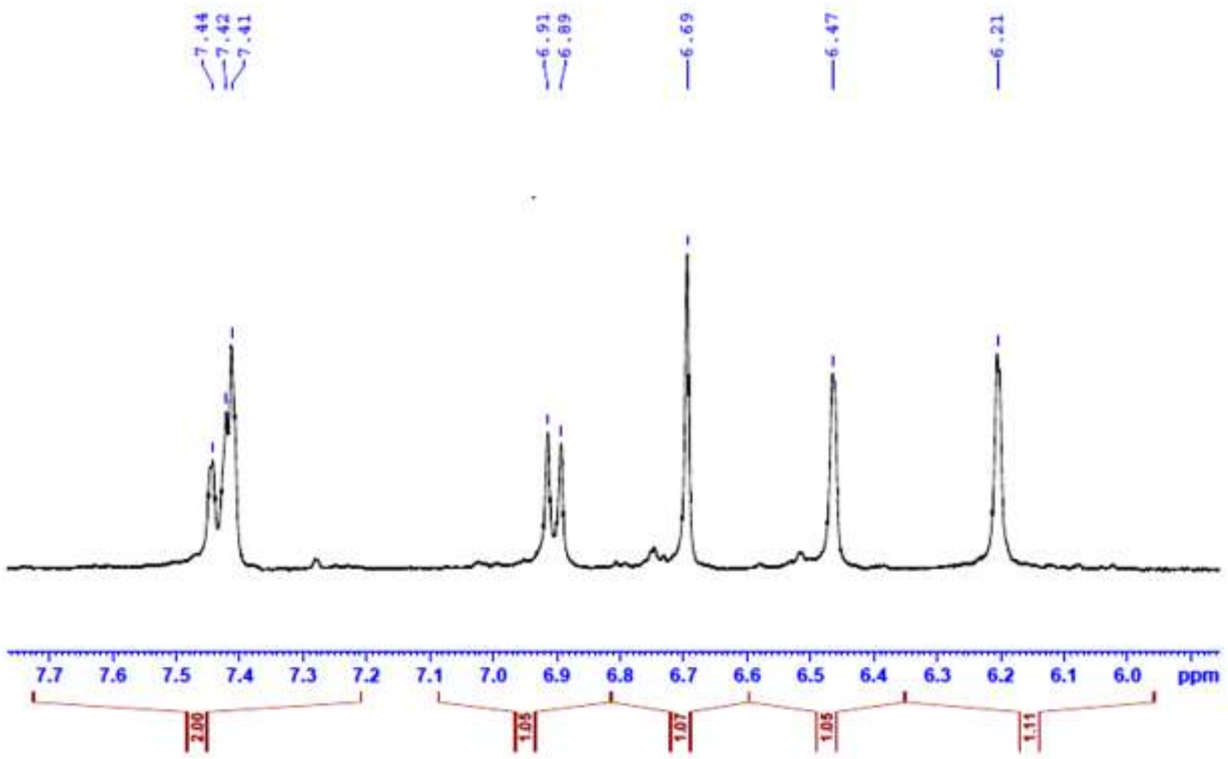

Figure S14. Partial expansion of ^{1}H-NMR (400 MHz, DMSO-d_6) spectrum of compound **4**

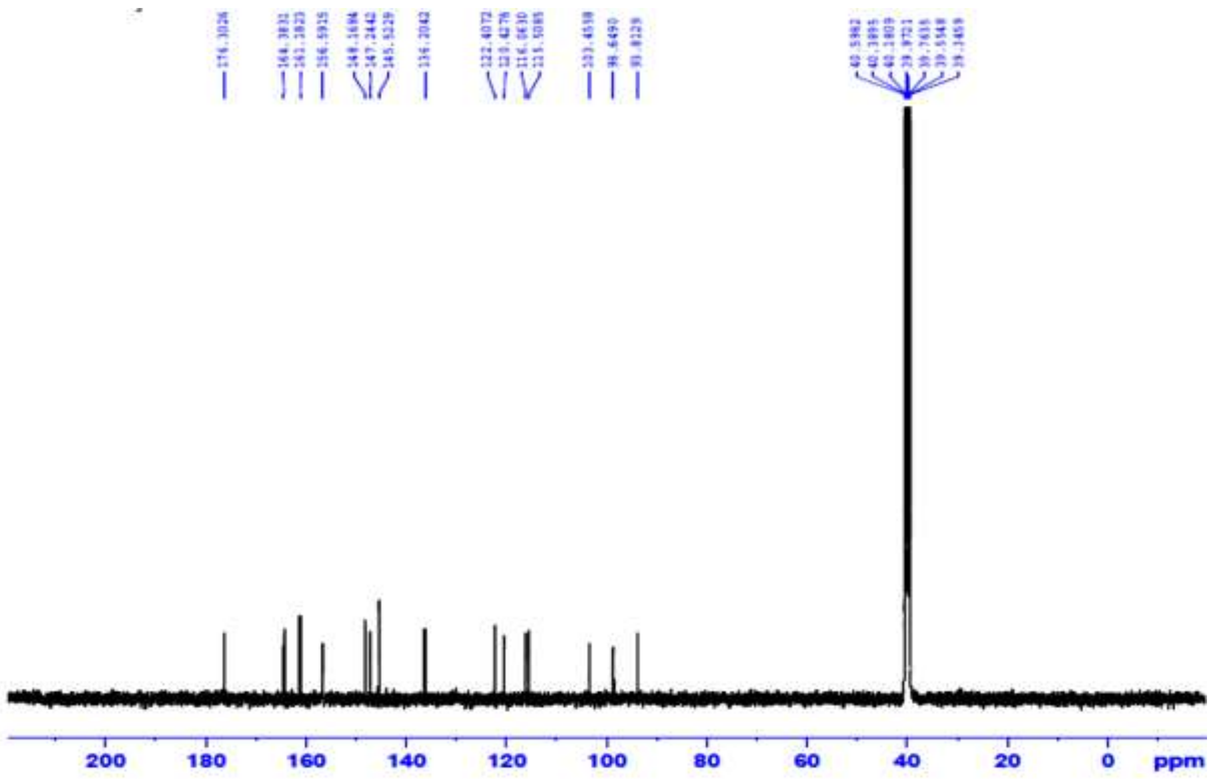

Figure S15. ^{13}C-NMR (100 MHz, DMSO-d_6) spectrum of compound **5**

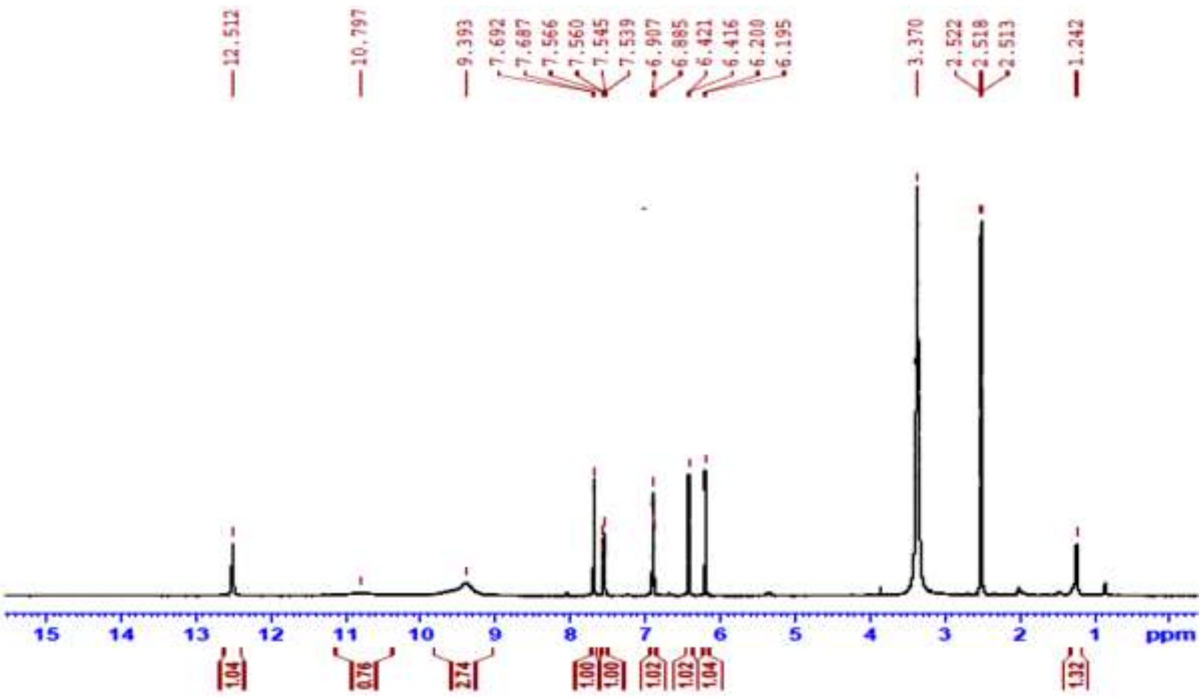

Figure S16. ^{1}H-NMR (400 MHz, DMSO-d_6) spectrum of compound **5**

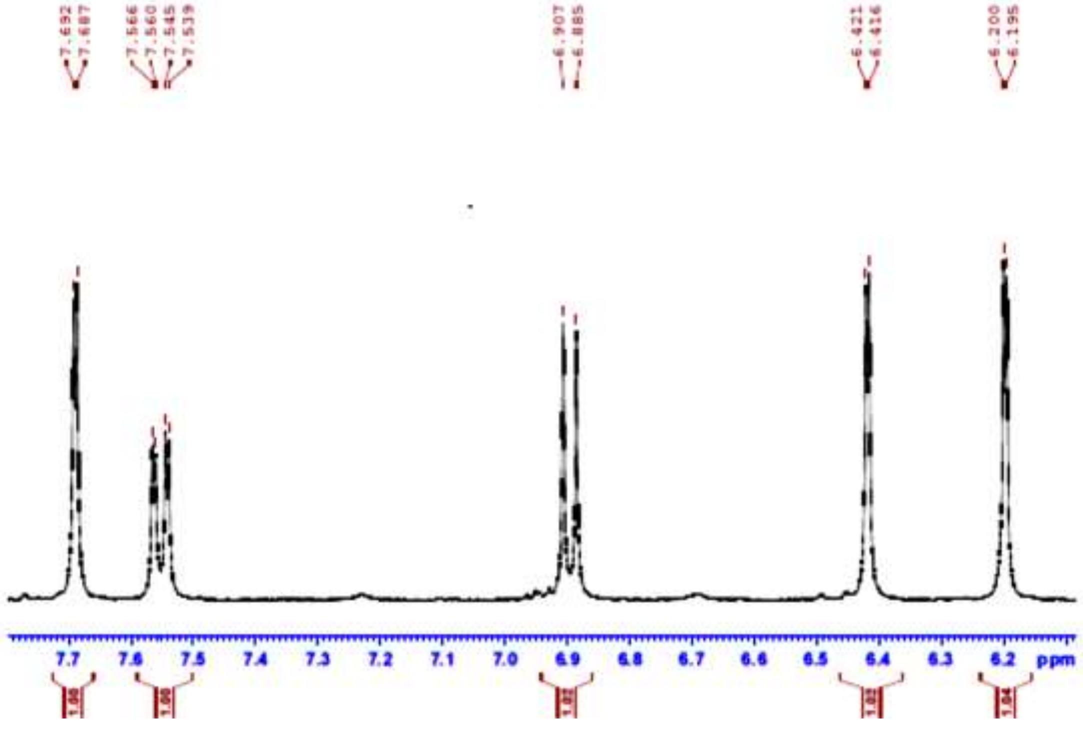

Figure S17. Partial expansion of ^{1}H-NMR (400 MHz, DMSO-d_6) spectrum of compound **5**

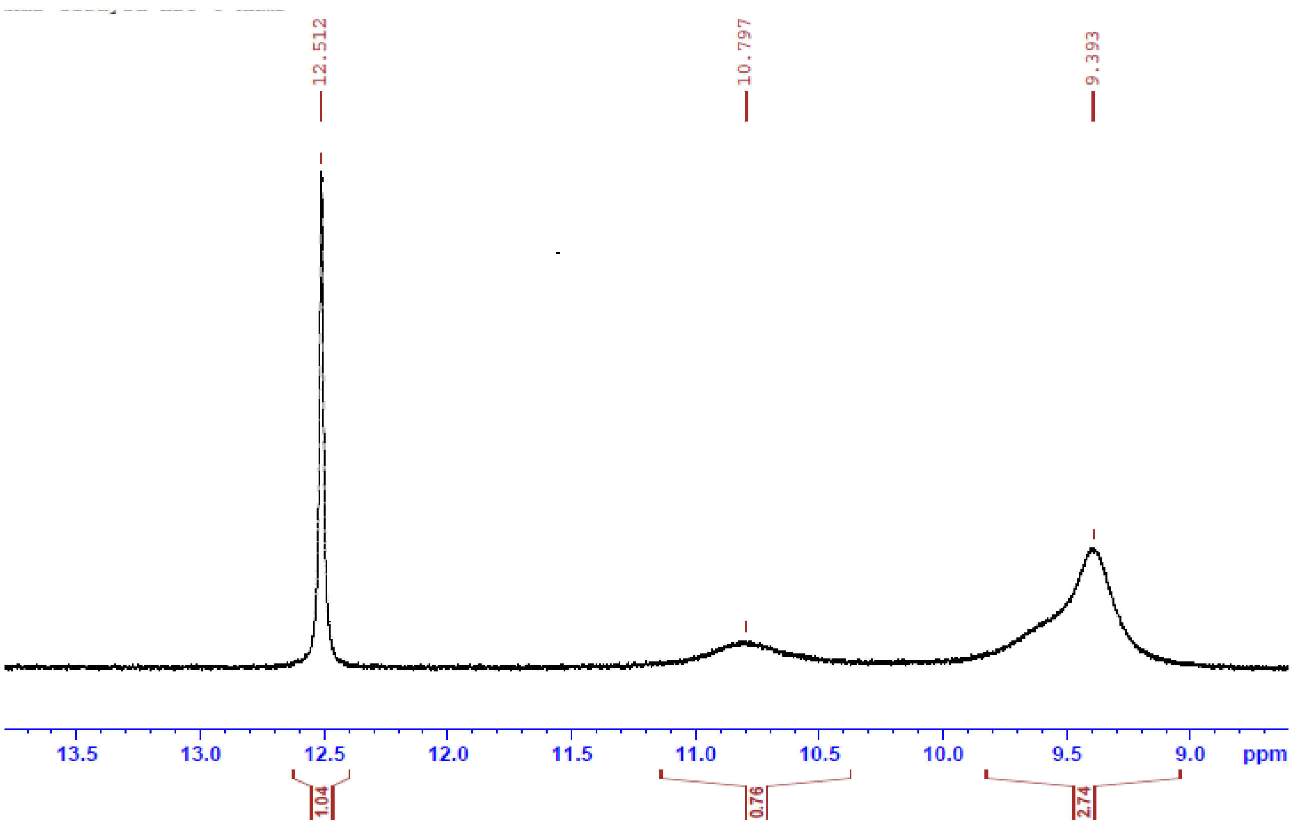

Figure S18. Partial expansion of ^{1}H-NMR (400 MHz, DMSO-d_6) spectrum of compound **5**

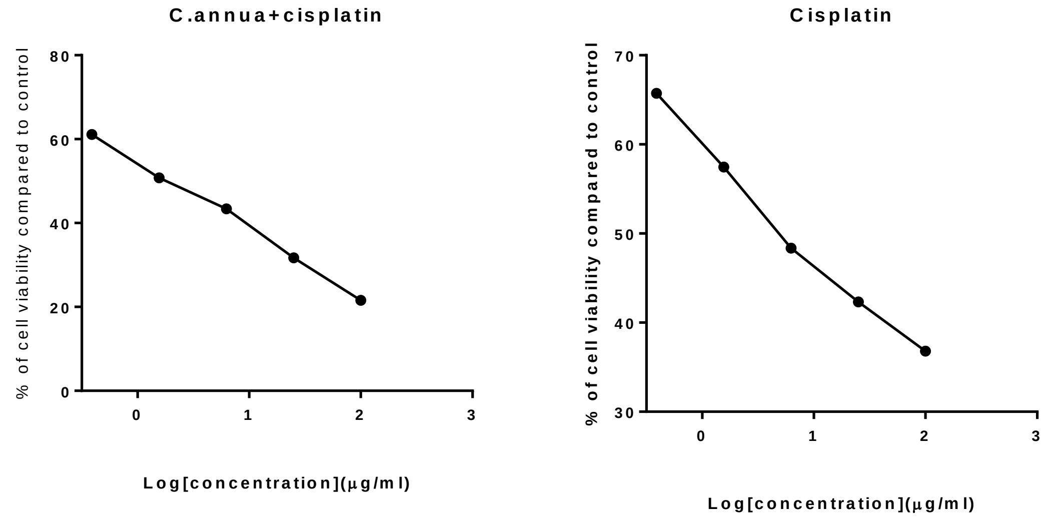

Figure S19. MTT assay dose-response curve of cisplatin and cisplatin - *C. annua* extract combination on MCF-7 cells

Data of Compound 1	
Trans-Ferulic acid (**1**)	m.p 169 °C; [1] H-NMR (400 MHz, CD_3OD): δ (ppm), 7.49 (1H, d, *J* = 15.6 Hz, H-7), 7.07 (1H, d, *J* = 2.0 Hz, H-2), 6.95 (1H, dd, *J* = 8.4, 2.0 Hz, H-6), 6.71 (1H, d, *J* = 8.4 Hz, H-5), 6.21 (1H, d, *J* = 16.0 Hz, H-8), 3.78 (3H, s, OCH_3); ^{13}C-NMR (CD_3OD, 100 MHz) δ (ppm), 126.4 (C-1), 110.2 (C-2), 149.1 (C-3), 148.0 (C-4), 115.0 (C-5), 122.6 (C-6), 145.5 (C-7), 114.5 (C-8), 169.6 (C-9).

Table S1. The ^{1}H and ^{13}C-NMR spectral data as well as the melting point of the isolated compound **1**

Data of Compound **2**	
Kaempferol (**2**)	m.p 286 °C; [1] H-NMR (400 MHz, CD_3OD): δ (ppm), 8.00 (2H, d, *J* = 8.8 Hz, H-2' and H-6'), 6.81 (2H, d, *J* = 8.8 Hz, H-3' and H-5'), 6.30 (1H, d, *J* = 2.0 Hz, H-8), 6.09 (1H, d, *J* = 2.0 Hz, H-6); ^{13}C-NMR (CD_3OD, 100 MHz) δ (ppm), 146.7 (C-2), 137.3 (C-3), 176.0 (C-4), 161.1 (C-5), 97.8 (C-6), 164.2 (C-7), 93.0 (C-8), 156.9 (C-9), 103.1 (C-10), 122.3 (C-1'), 129.3 (C-2'), 114.9 (C-3'), 159.2 (C-4'), 114.9 (C-5'), 129.3 (C-6').

Table S2. The ^{1}H and ^{13}C-NMR spectral data as well as the melting point of the isolated compound **2**

Data of Compound **3**	
p-Coumaric acid (**3**)	m.p 134 °C; 1 H-NMR (400 MHz, CD_3OD): δ (ppm), 7.50 (1H, d, *J* = 16.0 Hz, H-7), 7.34 (2H, d, *J* = 8.4 Hz, H-2 and H-6), 6.71 (2H, d, *J* = 8.8 Hz, H-3 and H-5), 6.18 (1H, d, *J* = 16.0 Hz, H-8); ^{13}C-NMR (CD_3OD, 100 MHz) δ (ppm), 126.1 (C-1), 129.7 (C-2), 115.4 (C-3), 159.8 (C-4), 115.4 (C-5), 129.7 (C-6), 145.3 (C-7), 114.2 (C-8), 169.7 (C-9).

Table S3. The ^{1}H and ^{13}C-NMR spectral data as well as the melting point of the isolated compound **3**

Data of Compound **4**	
Luteolin (**4**)	m.p 329 °C; ^{1}H-NMR (DMSO-d_6, 400 MHz): δ (ppm), 13.00 (1H, br. s, OH-5), 7.41-7.45 (2H, m, H-2' and H-6'), 6.90 (1H, d, *J* = 8.0 Hz, H-5'), 6.71 (1H, s, H-3), 6.46 (1H, s, H-8), 6.20 (1H, s, H-6); ^{13}C-NMR (DMSO-d_6, 100 MHz) δ (ppm), 165.0 (C-2), 103.2 (C-3), 182.1 (C-4), 162.0 (C-5), 99.4 (C-6), 164.3 (C-7), 94.4 (C-8), 157.8 (C-9), 104.0 (C-10), 121.8 (C-1'), 113.7 (C-2'), 146.3 (C-3'), 150.4 (C-4'), 116.5 (C-5'), 119.4 (C-6').

Table S4. The ^{1}H and ^{13}C-NMR spectral data as well as the melting point of the isolated compound **4**

Data of Compound **5**	
Quercetin (**5**)	m.p 317 °C; ^{1}H-NMR (DMSO-d_6, 400 MHz): δ (ppm), 12.51 (1H, br. s, OH-5), 6.20 (1H, d, J = 2.0 Hz, H-6), 10.80 (1H, s, OH-7), 6.42 (1H, d, J = 2.0 Hz, H-8), 7.69 (1H, d, J = 2.0 Hz, H-2'),9.39 (1H, br. s, OH-3'), 9.39 (1H, br. s, OH-4'), 6.90 (1H, d, J = 8.8 Hz, H-5'), 7.55 (1H, dd, J=8.4, 2.4 Hz, H-6'); ^{13}C-NMR (DMSO-d_6, 100 MHz) δ (ppm), 147.2 (C-2), 136.2 (C-3), 176.3 (C-4), 161.3 (C-5), 98.6 (C-6), 164.4 (C-7), 93.8 (C-8), 156.6 (C-9), 103.5 (C-10), 122.4 (C-1'), 115.5 (C-2'), 145.5 (C-3'), 148.2 (C-4'), 116.1 (C-5'), 120.4 (C-6').

Table S5. The ^{1}H and ^{13}C-NMR spectral data as well as the melting point of the isolated compound **5**

Compound	Structure	Ligand-receptor interactions towards caspase-3 (PDB=6CKZ)	Ligand-receptor interactions towards IFN-γ (PDB: 2R3Z)
Methanesulfonic acid	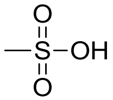	--	--
Caffeoyl-quinic acid	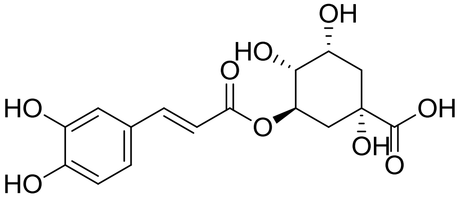	1HB with Arg 207<br>1HB with His 121<br>1 Arene-cation His 121	1 HB with Ile 12<br>1 HB with Asp 15<br>1 Arene-cation His 13
Glucotropaeolin	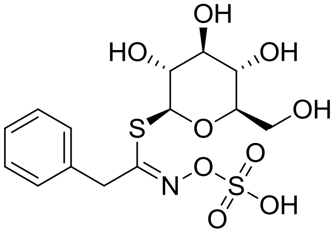	1HB with Arg 64<br>1HB with His 121<br>1 Arene-cation Trp 206	1 HB with Ile 12<br>1 HB with Cys 53
1H-indole-3 carboxylic acid	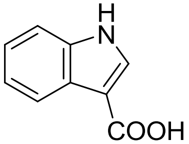	--	--
Progoitrin	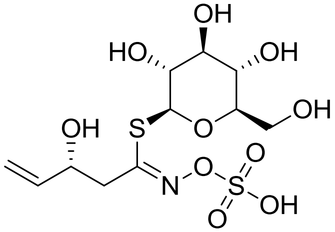	1HB with Arg 207<br>1HB with His 121<br>1 Arene-cation Arg 207	1 HB with Gln 51<br>1 HB with Asp 15<br>1 Arene-cation Ile 12
3-formylindole	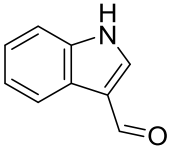	--	--

Quercetin-*3-O*-arabinoglucoside	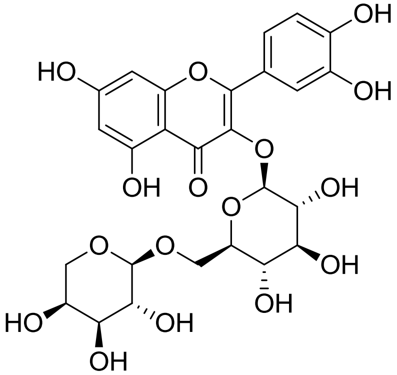	1HB with Arg 207<br>1HB with His 121	1 HB with Asp 15<br>1 Arene-cation Ile 12
Quercetin *3-O-β*-D-glucopyranosyl-(1→2)-arabinopyranoside	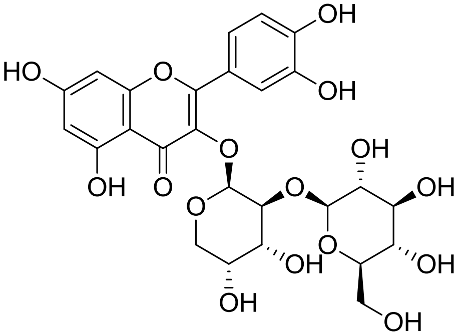	2HB with His 121<br>1 Arene-cation Arg 207	1 HB with Ile 12<br>1 HB with Cys 53
2-(1H-indol-3-yl) acetic acid	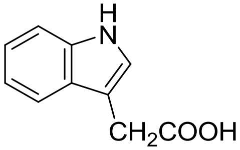	--	--
Sinigrin	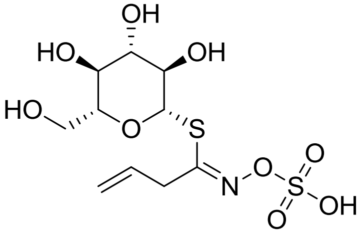	2HB with Arg 121<br>1HB with His 121	1 HB with Ile 12<br>1 Arene-cation His 13

Kaempferol 3, 7 di-glucoside	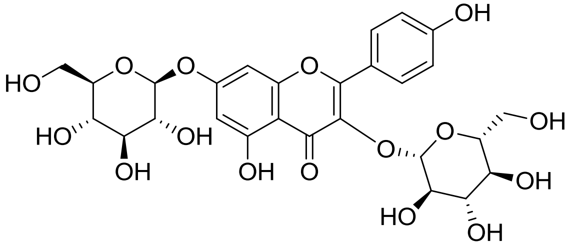	1HB with Arg 207<br>1HB with His 121	1 HB with His 13<br>1 HB with Cys 53
Quercetin*3-O*- [(6 sinapoyl-*β*-glucopyranosyl) (1→2)-*β*-arabinopyranoside]-*7-O*-*β*-glucopyranoside	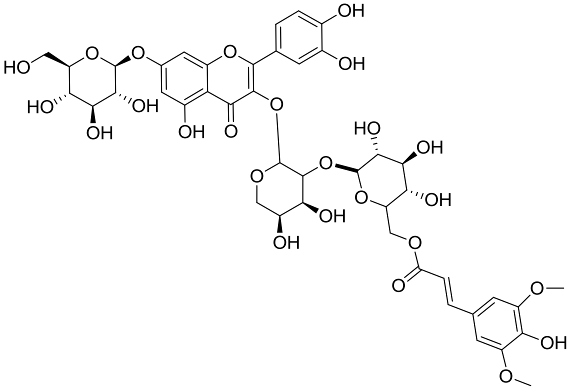	1HB with Arg 207<br>1HB with Arg 64	1 HB with Gln 51<br>1 HB with Asp 15<br>1 Arene-cation His 13
Quercetin*3-O*-[(6-feruloyl-*β*-glucopyranosyl) -(1→2)-*β*-arabinopyranoside]-*7-O*-*β*-glucopyranoside	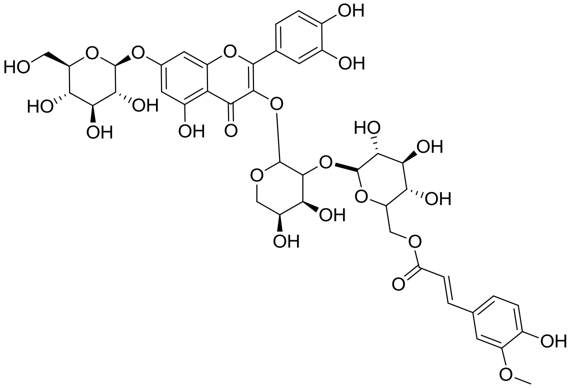	1HB with His 121	1 HB with Gln 51<br>1 HB with Asp 15<br>1 Arene-cation Ile 12
1-methoxy-1H-indole-3-carbaldehyde	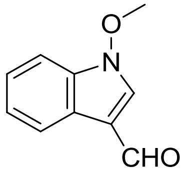	--	--

4-Hydroxyglucobrassicin	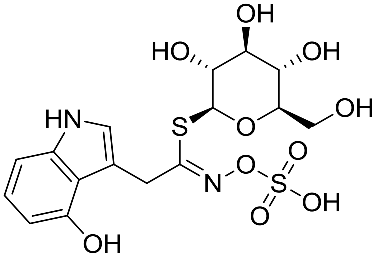	1HB with Gly 122<br>1HB with His 121	1 HB with Ile 12<br>1 Arene-cation Asp 15<br>1 Arene-cation His 13
9(methylsulfonyl)hydroxy nonyl glucosinolate	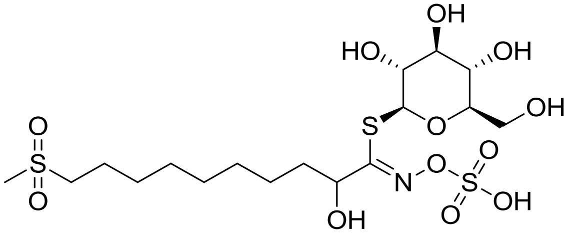	1HB with Gly 122<br>1HB with Arg 207	1 HB with Gln 51<br>1 HB with Cys 53<br>1 Arene-cation Ile 12
Kaempferol-3 rutinoside	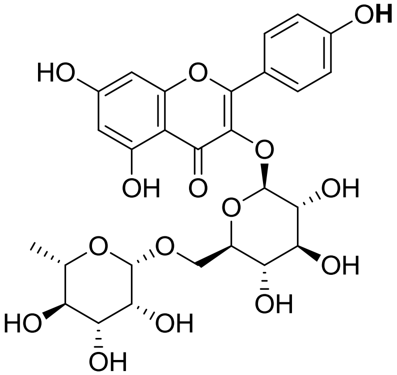	1 HB with His 121<br>1 HB with Arg 64<br>1 Arene-cation His 121	1 HB with His 13<br>1 HB with Gln 51<br>1 Arene-cation His 13
Syringaldehyde	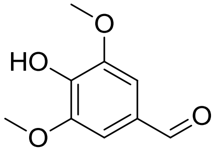	--	--
Quercetin-4'-glucoside	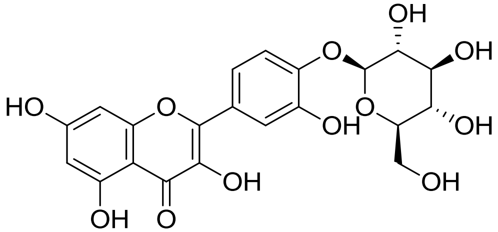	2 HB with Arg 207<br>1 Arene-cation Arg 207	1 HB with Ile 1<br>1 HB with Cys 53<br>1 Arene-cation Ile 12

Luteolin-7-O-glucoside	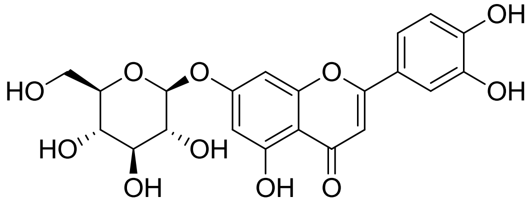	1 HB with His 121<br>1 Arene-cation His 121	1 HB with Gln 51<br>1 HB with Cys 53
Kaempferol-*3,7-O-bis-α*-L-rhamnoside	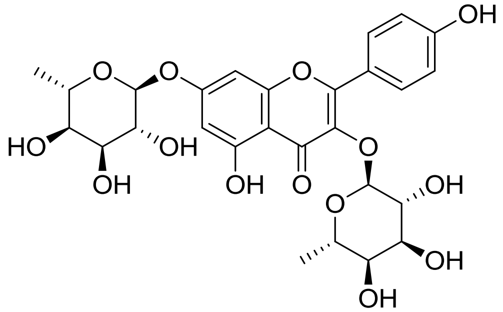	1 HB with Arg 207<br>1 HB with Arg 64	1 HB with His 13<br>1 Arene-cation His 13<br>1 HB with Asp 15
Quercetin *3-O*-galactoside	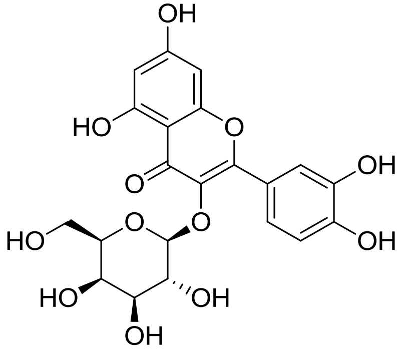	2 HB with Arg 207	1 HB with Gln 51<br>1 HB with Cys 53<br>1 Arene-cation Ile 12
Quercetin-3-D-xyloside	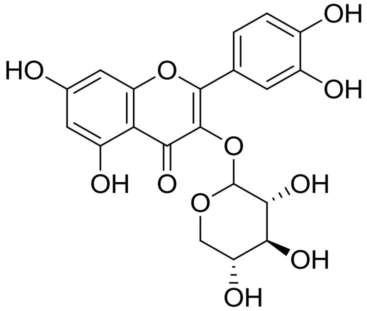	1 HB with His 121<br>1 Arene-cation His 121	1 HB with Ile 1<br>1 HB with Cys 53<br>1 Arene-cation His 13

Cyanidin-3-glucoside	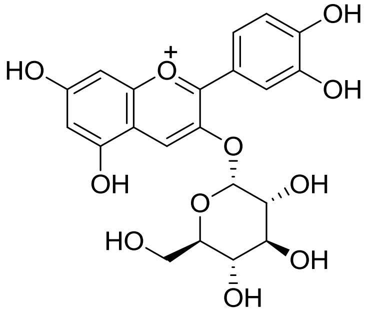	1 HB with Arg 207<br>I HB with His 121	1 HB with Gln 51<br>1 HB with Cys 53<br>1 HB with Asp 15
Kaempferol-3-*O* glucoside	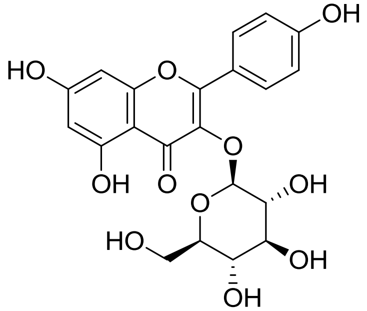	2 HB with Arg 64	1 HB with Cys 53<br>1 HB with Cys 53<br>1 Arene-cation Ile 12
Quercetin *3-O*-[(6-sinapoyl-*β*-glucopyranosyl) -(1→2)-*β* arabinopyranoside	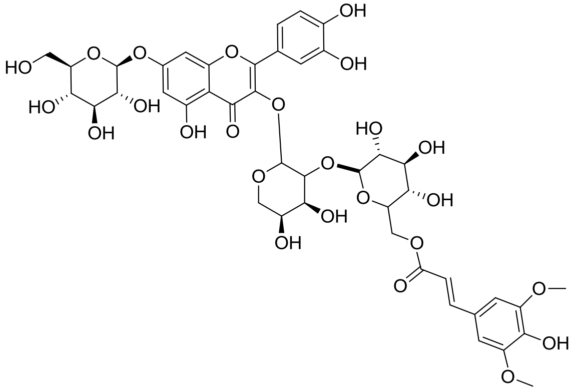	1 HB with Arg 207<br>1 HB with His 121<br>1 Arene-cation His 121	1 HB with Ile 1<br>1 HB with Cys 53<br>1 Arene-cation His 13

Petunidin-*3-O-β*-glucopyranoside	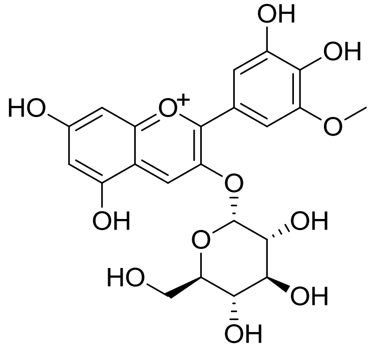	2 HB with His 121	1 HB with Gln 51<br>1 HB with Asp 15<br>1 Arene-cation His 13
Vitexin	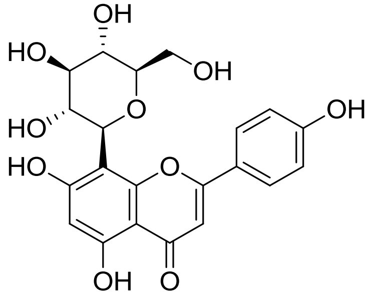	1 HB with His 121<br>I HB with Arg 207	1 HB with Cys 53<br>1 HB with Asp 15
Cosmosiin	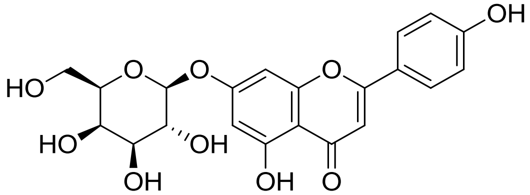	2 HB with Arg 64<br>1 Arene-cation Arg 64	1 HB with Gln 51<br>1 HB with Cys 53
Syringetin-*3-O*-glucoside	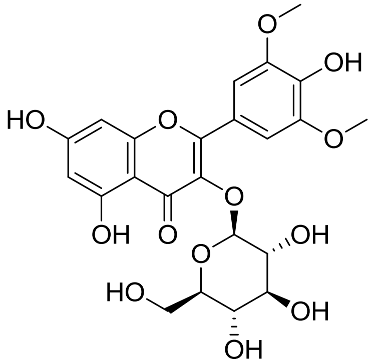	2 HB with His 121<br>1 Arene-cation his 121	1 HB with Gln 51<br>1 HB with Cys 53<br>1 Arene-cation His 13

Peonidine-*3*-*O*-glucoside	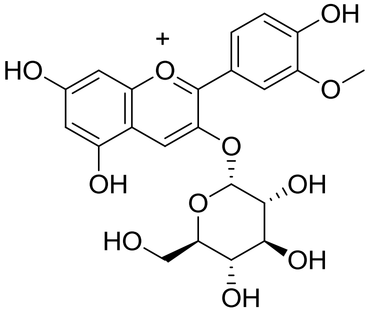	1 HB with His 121<br>1 HB with Arg 207<br>1 Arene-cation his 121	1 HB with Cys 53<br>1 Arene-arene His 13
Malvidin-3-galactoside	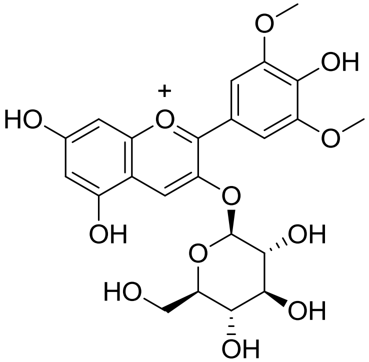	1 HB with Arg 207<br>1 HB with Arg 64	1 HB with Ile 12<br>1 HB with Asp 15<br>1 Arene-cation His 13
Caffeic acid	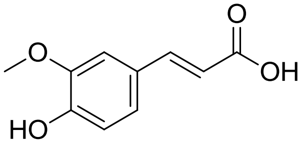	--	--
Hesperetin	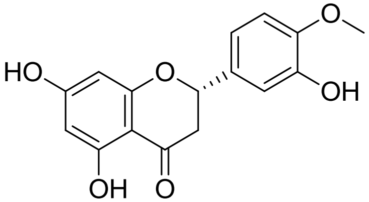	1 HB with Arg 64<br>1 HB with Arg 207<br>1 Arene-cation Arg 207	1 HB with Gln 51<br>1 HB with Cys 53
Isorhamnetin	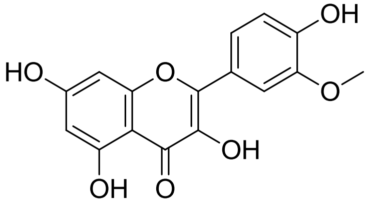	1 HB with His 121<br>1 HB with Arg 207<br>1 Arene-arene Trp 206	--

Sinapic acid	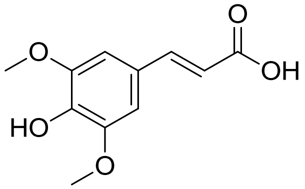	1 HB with His 121<br>1 HB with Arg 207<br>1 Arene-arene Trp 206	--
Apigenin	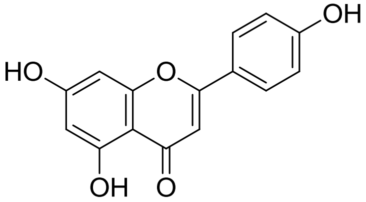	1 HB with His 121<br>1 HB with Arg 64	1 HB with Gln 51<br>1 HB with Asp 15
Kaempferide	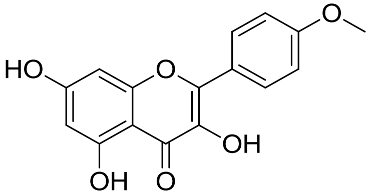	1 HB with His 121<br>1 HB with Arg 207<br>1 Arene-cation Arg 207	1 HB with Ile 12<br>1 HB with Cys 53

Table S6. Summary of ligand-receptor interactions of the previously identified compounds in *C. annua* extract towards caspase-3 (PDB=6CKZ) and IFN-γ (PDB: 2R3Z)
